# The BAF complex inhibitor pyrimethamine reverses HIV-1 latency in people with HIV-1 on antiretroviral therapy

**DOI:** 10.1126/sciadv.ade6675

**Published:** 2023-03-15

**Authors:** Henrieke A. B. Prins, Raquel Crespo, Cynthia Lungu, Shringar Rao, Letao Li, Ronald J. Overmars, Grigorius Papageorgiou, Yvonne M. Mueller, Mateusz Stoszko, Tanvir Hossain, Tsung Wai Kan, Bart J. A. Rijnders, Hannelore I. Bax, Eric C. M. van Gorp, Jan L. Nouwen, Theodora E. M. S. de Vries-Sluijs, Carolina A. M. Schurink, Mariana de Mendonça Melo, Els van Nood, Angela Colbers, David Burger, Robert-Jan Palstra, Jeroen J. A. van Kampen, David A. M. C. van de Vijver, Thibault Mesplède, Peter D. Katsikis, Rob A. Gruters, Birgit C. P. Koch, Annelies Verbon, Tokameh Mahmoudi, Casper Rokx

**Affiliations:** ^1^ Department of Internal Medicine, Section Infectious Diseases, Erasmus University Medical Center, Rotterdam, Netherlands.; ^2^ Department of Medical Microbiology and Infectious Diseases, Erasmus University Medical Center, Rotterdam, Netherlands.; ^3^ Department of Biochemistry, Erasmus University Medical Center, Rotterdam, Netherlands.; ^4^ Department of Pharmacy, Erasmus University Medical Center, Rotterdam, Netherlands.; ^5^ Department of Viroscience, Erasmus University Medical Center, Rotterdam, Netherlands.; ^6^ Department of Biostatistics, Erasmus University Medical Center, Rotterdam, Netherlands.; ^7^ Department of Immunology, Erasmus University Medical Center, Rotterdam, Netherlands.; ^8^ Department of Pathology, Erasmus University Medical Center, Rotterdam, Netherlands.; ^9^ Department of Urology, Erasmus University Medical Center, Rotterdam, Netherlands.; ^10^ Department of Pharmacy, Radboud Institute for Health Sciences, Radboud University Medical Center Nijmegen, Nijmegen, Netherlands.; ^11^ Department of Internal Medicine, University Medical Center, Utrecht, Netherlands.

## Abstract

Reactivation of the latent HIV-1 reservoir is a first step toward triggering reservoir decay. Here, we investigated the impact of the BAF complex inhibitor pyrimethamine on the reservoir of people living with HIV-1 (PLWH). Twenty-eight PLWH on suppressive antiretroviral therapy were randomized (1:1:1:1 ratio) to receive pyrimethamine, valproic acid, both, or no intervention for 14 days. The primary end point was change in cell-associated unspliced (CA US) HIV-1 RNA at days 0 and 14. We observed a rapid, modest, and significant increase in (CA US) HIV-1 RNA in response to pyrimethamine exposure, which persisted throughout treatment and follow-up. Valproic acid treatment alone did not increase (CA US) HIV-1 RNA or augment the effect of pyrimethamine. Pyrimethamine treatment did not result in a reduction in the size of the inducible reservoir. These data demonstrate that the licensed drug pyrimethamine can be repurposed as a BAF complex inhibitor to reverse HIV-1 latency in vivo in PLWH, substantiating its potential advancement in clinical studies.

## Teaser

The antiprotozoal pyrimethamine reactivates latent HIV in vivo and can be used in the search for an HIV cure.

## INTRODUCTION

Despite markedly improved treatment prospects brought about by combination antiretroviral therapy (cART), an infection with HIV-1 remains a chronic condition that, with few exceptions, necessitates lifelong cART. A pool of long-lived CD4^+^ T cells harbors latent, replication-competent provirus integrated in the genome. This latent HIV-1 reservoir is established in the first weeks after HIV-1 infection and persists stably even after prolonged periods of cART (*1*). Treatment cessation results in rapid plasma viral rebound causing disease progression in people living with HIV-1 (PLWH) (*2*, *3*). Therefore, interventions beyond cART are needed toward an HIV-1 cure.

One of the key approaches currently proposed to eradicate the latent HIV-1 reservoir in PLWH is to pharmacologically reverse latency via latency reversing agents (LRAs). Elimination of the reactivated reservoir cells could then be triggered by intrinsic proapoptotic pathways or via extracellular immune cell–mediated mechanisms (*4*–*6*). The ultimate aim is to accomplish a prolonged state of sustainable viral remission without cART in PLWH.

The multitude of LRAs that have been identified through mechanism-based approaches or large-scale screening of compound libraries can be categorized based on distinct molecular and pharmacological targets (*7*, *8*), all leading to reactivation of HIV-1 transcription. The LRA classes that influence this process can be classified as derepressors antagonizing the repressive latent HIV-1 promotor locus, or as activators that directly stimulate viral transcription initiation or elongation. The importance of blocks at the posttranscriptional level has also come to light recently and may be targetable by LRAs (*9*–*12*). In contrast to the numbers identified in vitro, only a few LRAs have regulatory approval and can currently be used in clinical settings. The deacetylation of histones has been identified and extensively studied as a major mechanism to promote HIV-1 latency (*13*–*15*), and histone deacetylase inhibitors (HDACis) have become the main evaluated LRA class in HIV-1 cure clinical trials (*16*). The first case series used the HDACi valproic acid (VPA) and showed an impact on the reservoir of PLWH who also received intensified cART (*17*). However, subsequent larger clinical studies using VPA as HDACi without intensified cART did not demonstrate a reduction in the size of the reservoir (*18*–*22*). Other studies were conducted with clinically approved HDACis that were repurposed as LRA including vorinostat (*23*–*33*), panobinostat (*34*), and romidepsin (*35*–*39*) and showed that, in general, HDACis can safely induce HIV-1 transcription at tolerable concentrations in PLWH. However, no HDACi led to a significant reduction of the proviral reservoir when used alone. Studies on the limited number of other LRA classes explored in PLWH have resulted in similar conclusions (*40*–*43*). Cumulative evidence therefore indicates the need for identification of previously unidentified LRA classes that potently reactivate HIV-1 and induce reservoir eradication and/or to use combinatorial LRA approaches to reach the potency required to obtain a significant impact on the size of the replication competent reservoir.

Our preclinical studies identified BRG–Brahma associated factor (BAF) complex inhibitors (BAFi) as a novel LRA class, which we demonstrated enhanced the activity of other LRA classes (*44*–*46*). The BAF (mammalian SWI/SNF) chromatin remodeling complex is a key repressor of HIV-1 latency that uses adenosine triphosphate (ATP) hydrolysis to position repressive nucleosome nuc-1 at the HIV-1 long-terminal repeat, causing transcriptional silencing of HIV-1 gene expression, thereby maintaining HIV-1 latency (*47*, *48*). The pharmacological inhibition of the BAF complex leads to derepression of HIV-1 transcription, resulting in latency reversal (*44*, *45*). The clinically licensed drug pyrimethamine was subsequently identified as a potent BAFi to reverse HIV-1 latency in primary cell models of latency and in cells obtained from PLWH on cART at tolerable concentrations for humans (*44*). Pyrimethamine also enhanced the activity of other LRAs, including HDACis, in cell lines and primary cell models of HIV-1 latency (*44*). Pyrimethamine is an orally administered, inexpensive antiprotozoan drug widely used in humans, including in people with AIDS, rendering pyrimethamine an attractive candidate for LRA studies (*49*–*57*).

To study the efficacy of BAF complex inhibition by pyrimethamine to reactivate the HIV-1 reservoirs in PLWH, we conducted a proof-of-concept, randomized controlled clinical trial [LRAs United as a Novel Anti-HIV strategy (LUNA), clinicaltrials.gov identifier: NCT03525730]. Apart from a monotherapy arm, we designed a parallel arm where pyrimethamine was combined with an HDACi as partner drug to study an all-oral, affordable, generic combination of LRAs. In contrast to previous studies, we chose the HDACi VPA at maximized dose, in an acid-resistant enteric formulation for better gastrointestinal drug absorption (*18*–*22*). While the previous clinical studies using VPA alone did not show a reduction in the reservoir size in PLWH, VPA had thus far never been used at optimized dosing or in combination with other LRAs (*18*–*22*). Our previous study showed that VPA was able to synergize in reactivating latent HIV-1 with (not yet clinically advanced) drugs from the BAF inhibitor class (*45*), in line with other data indicating activity of VPA as LRA (*23*, *58*, *59*). We therefore reasoned that this optimized dosing of VPA may enhance the effect of pyrimethamine additively or synergistically in vivo. We therefore designed the study to examine whether the activity of the individual LRAs can be potentiated in vivo. We enrolled participants on suppressive cART and randomized them to 14 days of pyrimethamine (200 mg once daily at day 1, then 100 mg once daily), VPA (30 mg/kg per day in two equal doses), pyrimethamine and VPA, or control with a 4-week follow-up after treatment to study clinical safety and efficacy. Our findings demonstrate that pyrimethamine reactivated HIV-1 in vivo in PLWH on suppressive cART, supporting its further development into clinical strategies to target the latent reservoir for reactivation. Despite reversal of latency, pyrimethamine treatment did not lead to a reduction in the size of the inducible reservoir.

## RESULTS

### Combination therapy led to treatment adjustments but no serious adverse events

**Fig. 1.**
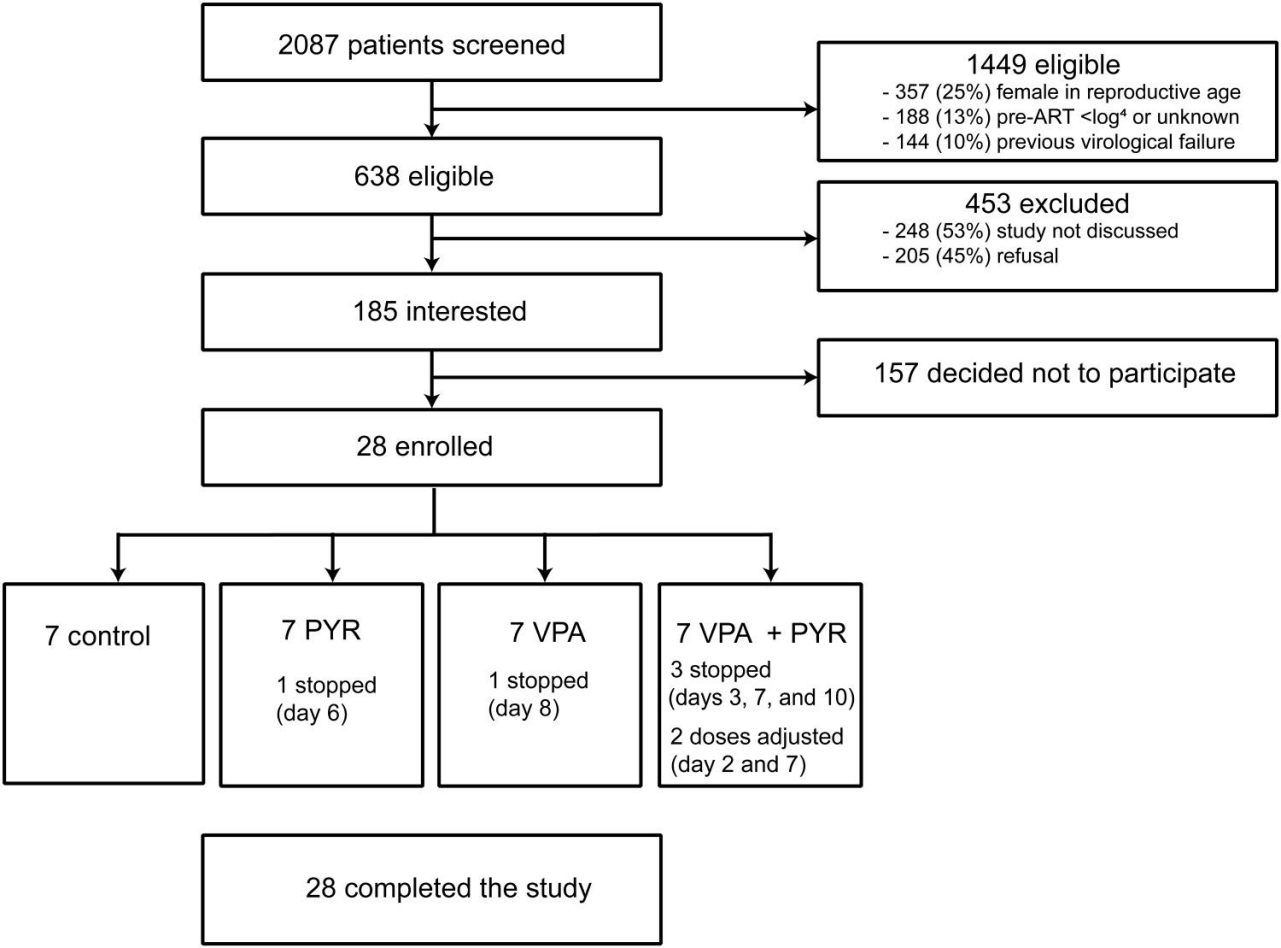
Study flow diagram showing the information about the method of recruitment and the number of participants that completed the LUNA study. Miscellaneous category for ineligibility covers not fitting the other study inclusion and exclusion criteria, clinical judgment of treating physician, and study not discussed. ART, antiretroviral therapy; PYR, pyrimethamine.

Twenty-eight individuals living with HIV-1 on suppressive cART were enrolled and randomized into four arms of seven participants including three interventional arms and one control arm (Fig. 1). All participants continued daily cART throughout the study. Participants were men, predominantly white European, and on cART for a median of 7.5 years [interquartile range (IQR), 5.6 to 11.7] with a median CD4^+^ T cell count of 665 cells/μl (IQR, 530 to 820) at inclusion (Table 1). Integrase inhibitor–based cART regimens were used by 43% of participants; the remainder used non-nucleoside reverse transcriptase inhibitor–based cART. Baseline characteristics were comparable between the study arms including the median duration of therapy.

**Table 1.** Baseline characteristics of the participants at inclusion. Data are number with percentage or median with IQRs. INSTI, integrase strand transfer inhibitor; NNRTI, non-nucleoside reverse transcriptase inhibitor; PYR, pyrimethamine.

Baseline characteristics	Overall	PYR	VPA	PYR + VPA	Control
	*N* = 28	*n* = 7	*n* = 7	*n* = 7	*n* = 7
Male	28 (100)	7 (100)	7 (100)	7 (100)	7 (100)
Ethnic origin					
White European	24 (86)	4 (43)	7 (100)	6 (86)	7 (100)
Latin American or Hispanic	3 (11)	3 (57)	0	0	0
Black Caribbean	1 (4)	0	0	1 (14)	0
Age (years)	54 (47–61)	53 (48–55)	51 (54–67)	51 (45–58)	55 (51–64)
HIV subtype B*	24 (85.7)	7 (100)	6 (86)	6 (86)	5 (71)
History of AIDS	8 (29)	2 (29)	1 (14)	3 (43)	2 (29)
Years from HIV diagnosis until inclusion	8.5 (7.5–15.4)	7.7 (7.5–14.5)	7.8 (7.6–12.8)	7.6 (5.2–9.4)	15.6 (10.5–20.2)
Years on cART	7.5 (5.6–11.7)	7.3 (6.4–12.0)	7.6 (6.1–10.8)	5.6 (4.7–7.4)	9.8 (8.0–13.0)
Years with HIV-RNA <50 copies per ml	6.8 (4.9–11.1)	6.7 (5.1–11.5)	7.2 (5.4–9.5)	5.1 (4.5–7.1)	7.2 (5.2–12.5)
Initiated cART during acute HIV infection	3 (11)	2 (29)	1 (14)	0	0
Pre-cART plasma viral load zenith log_10_ copies per ml	4.9 (4.7–5.3)	5.3 (4.9–5.6)	4.8 (4.6–5.0)	4.9 (4.8–5.0)	4.9 (4.7–5.4)
Pre-cART nadir CD4^+^ T cell count per μl	235 (160–315)	290 (135–310)	250 (225–295)	240 (175–420)	210 (170–225)
CD4^+^ T cell count per μl at inclusion	665 (530–820)	610 (490–770)	750 (560–810)	630 (475–1005)	680 (590–740)
cART					
NNRTI-based†	16 (57)	5 (71)	4 (57)	4 (57)	3 (43)
INSTI-based‡	12 (43)	2 (29)	3 (43)	3 (43)	4 (57)

*Other HIV-1 subtypes include CRF01-AE (*n* = 2), CRF01-AG (*n* = 1), and C (*n* = 1).

†NNRTIs were rilpivirine (*n* = 6), nevirapine (*n* = 6), and efavirenz (*n* = 4). Fifteen NRTI backbones consisted of emtricitabine (FTC) and either tenofovir disoproxil fumarate (TDF) or tenofovir alafenamide (TAF), and 1 NRTI backbone consisted of abacavir with lamivudine.

‡INSTIs were dolutegravir (*n* = 10) and elvitegravir/cobicistat (*n* = 2). Seven NRTI backbones consisted of FTC and either TDF or TAF, three NRTI backbones consisted of lamivudine (3TC) and abacavir (ABC), and two had dolutegravir with 3TC as NRTI backbone.

During the 6-week study duration after inclusion, a total of 103 adverse events (AEs) were reported by the trial participants (tables S1 and S2). Except for three grade 3 AE (two with pyrimethamine alone and one in the combination arm), all were of grade 2 or lower severity (table S3). Most AEs (84 of 103) were experienced on pyrimethamine at a comparable rate between the monotherapy and the combination arms. Neurological and gastrointestinal symptoms were the most common AE categories across all interventional arms, with nausea, vomiting, and headache being the most frequently reported. No serious AEs (SAEs) were observed during the trial, and all AEs were resolved after the intervention. No AIDS-defining illnesses occurred during or after the study. The blood CD4^+^ T cell counts remained stable during the study without CD4^+^ T cell counts below the predefined safety limit of 200/μl. However, reported grade 1 and 2 AEs in seven of the participants necessitated the trial physicians to adjust the allocated treatment regimens. The PLWH with and without dose adjustments had a largely comparable clinical profile (table S4). Three participants in the combination arm stopped treatment at day 3 (LUNA-23), day 7 (LUNA-06), and day 10 (LUNA-21); one participant in the pyrimethamine arm stopped treatment at day 6 (LUNA-10) for a grade 1 AE; and one participant in the VPA arm stopped treatment at day 8 (LUNA-09). In one participant of the combination arm, we halved pyrimethamine dosage at day 7 (LUNA-12), while in another participant in the combination treatment arm we halved the VPA dose at day 2 and pyrimethamine dose at day 7 (LUNA-25) due to drug-related AE. These events stayed below the upper limit of our predefined safety criteria where we would stop the trial if more than two PLWH in a treatment arm had to discontinue treatment when 50% inclusion was reached, or if at any moment in the trial more than five PLWH had to discontinue their treatment for drug-related AE. We concluded that there was sufficient clinical-scientific and medical ethical basis to complete the study and had 100% follow-up of all 28 participants.

### Pyrimethamine induces HIV-1 transcription in vivo

To assess the potential of HIV-1 latency reversal in PLWH induced by pyrimethamine and VPA alone and in combination, we measured cell-associated unspliced (CA US) HIV-1 RNA by reverse transcription quantitative polymerase chain reaction (RT-qPCR) in CD4^+^ T cells isolated from peripheral blood at baseline (day 0, *t* = 0 hours), 6 hours after initiating the intervention regimen (day 0, *t* = 6 hours), at the end of the 2-week intervention phase (day 14), and 4 weeks thereafter (day 42) (fig. S1). Because of low cell numbers in the baseline sample, LUNA-06 (combination arm, participant stopped at day 7) was excluded from this primary end point analysis.

**Fig. 2.**
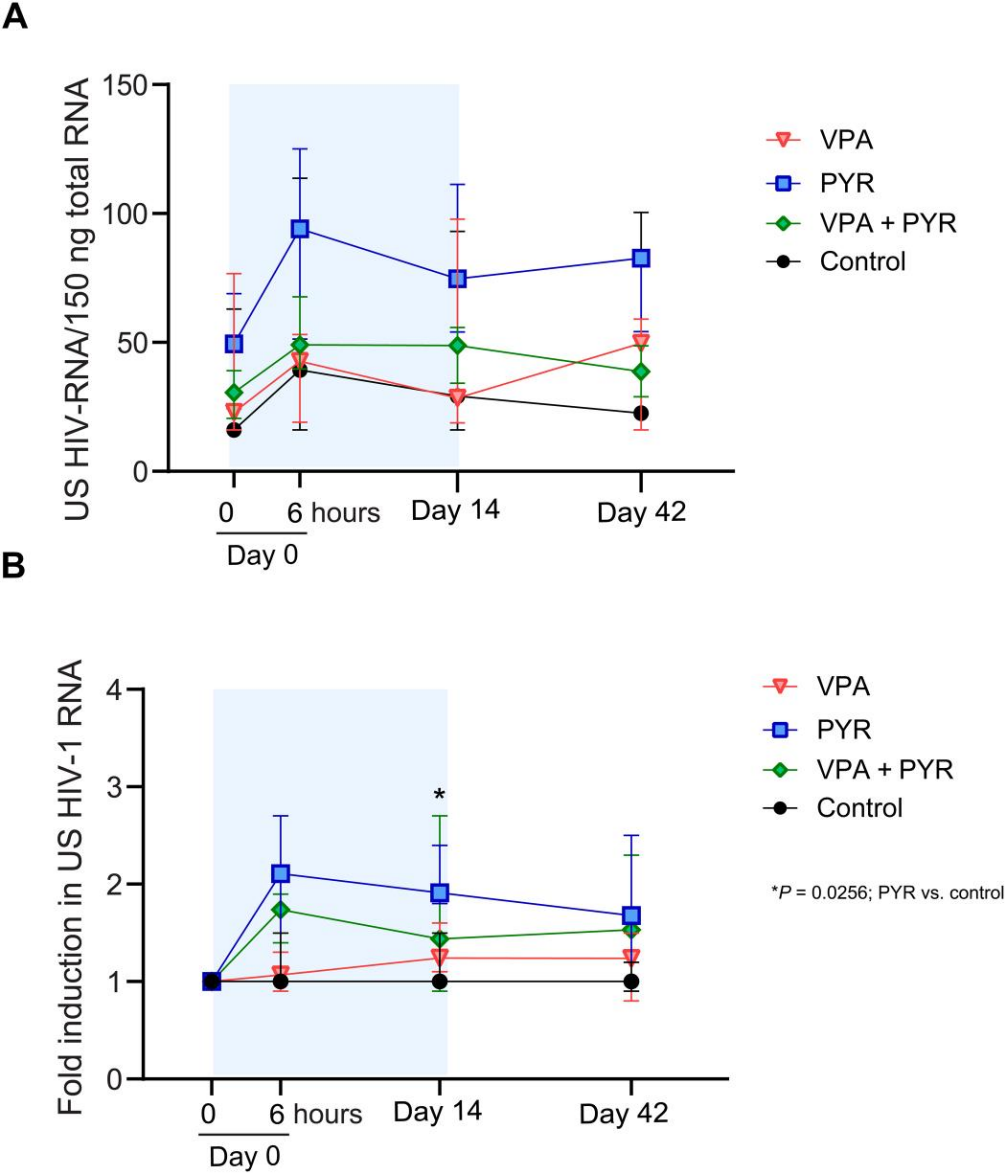
Changes in CA HIV-1 US RNA per arm at four time points during the LUNA study. (**A**) Median (IQR) total CA US HIV-1 RNA per 150 ng of total RNA in all four study arms at treatment initiation (day 0, *t* = 0 hours) and 6 hours after first dosing (day 0 *t* = 6 hours), at the end of the treatment period (day 14), and 28 days after the end of treatment (day 42). (**B**) Median (IQR) fold change in CA US HIV-1 RNA in all four study arms relative to baseline (day 0, *t* = 0 hours). * indicates the effect comparisons at the time points of the treatment arms compared to controls with *P* < 0.05.

Addressing the primary end point of the study, we found that, overall, the absolute change in CA US HIV-1 RNA from baseline during the 6-week study period between the treatment groups was different (*P* < 0.001) (Fig. 2A). This overall analysis cannot be used to conclude which groups or time points determined the effect, but the graphical data suggested a relevant contribution of the groups exposed to pyrimethamine. We performed additional analyses to substantiate this possibility. Overall, the fold change in CA US HIV-1 RNA is significantly induced in the pyrimethamine arm at day 14 both compared to day 0 pretreatment and compared to the control arm (Fig. 2B, Table 2, and table S5). Induction of CA US HIV-1 RNA in the pyrimethamine arm was consistently more prominent compared to controls, although not reaching statistical significance at all time points, likely due to the sample size (Table 2).

**Table 2.** The median fold change in CA US HIV-1 RNA per time point and per treatment arm compared to the control arm.

Treatment arm	Time point	Median fold change (normalized to day 0, 0 hours)	Interquartile range	*P* value compared to control arm*	*P* value within arm compared to day 0, 0 hours†
Control	0 *t* = 0 hours	1	1.0–1.0	–	–
	0 *t* = 6 hours	1	1.0–1.5	–	*P* = 0.14
	14	1	1.0–1.5	–	*P* = 0.27
	42	1	0.9–1.2	–	*P* = 0.47
Pyrimethamine	0 *t* = 0 hours	1	1.0–1.0	–	
	0 *t* = 6 hours	2.1073	1.5–2.7	*P* = 0.140	*P* = 0.028
	14	1.9129	1.8–2.4	*P* = 0.026	*P* = 0.018
	42	1.6775	1.2–2.5	*P* = 0.109	*P* = 0.043
Valproic acid	0 *t* = 0 hours	1	1.0–1.0	–	
	0 *t* = 6 hours	1.0667	0.9–1.3	*P* = 0.698	*P* = 0.46
	14	1.2401	1.1–1.6	*P* = 0.518	*P* = 0.12
	42	1.2377	0.8–1.5	*P* = 0.698	*P* = 0.25
Pyrimethamine + valproic acid	0 *t* = 0 hours	1	1.0–1.0	–	
	0 *t* = 6 hours	1.7396	1.4–1.9	*P* = 0.315	*P* = 0.046
	14	1.4372	0.9–2.7	*P* = 0.473	*P* = 0.12
	42	1.5312	1.0–2.3	*P* = 0.340	*P* = 0.27

*Mann-Whitney *U* tests.

†Wilcoxon signed-rank sum test.

**Fig. 3.**
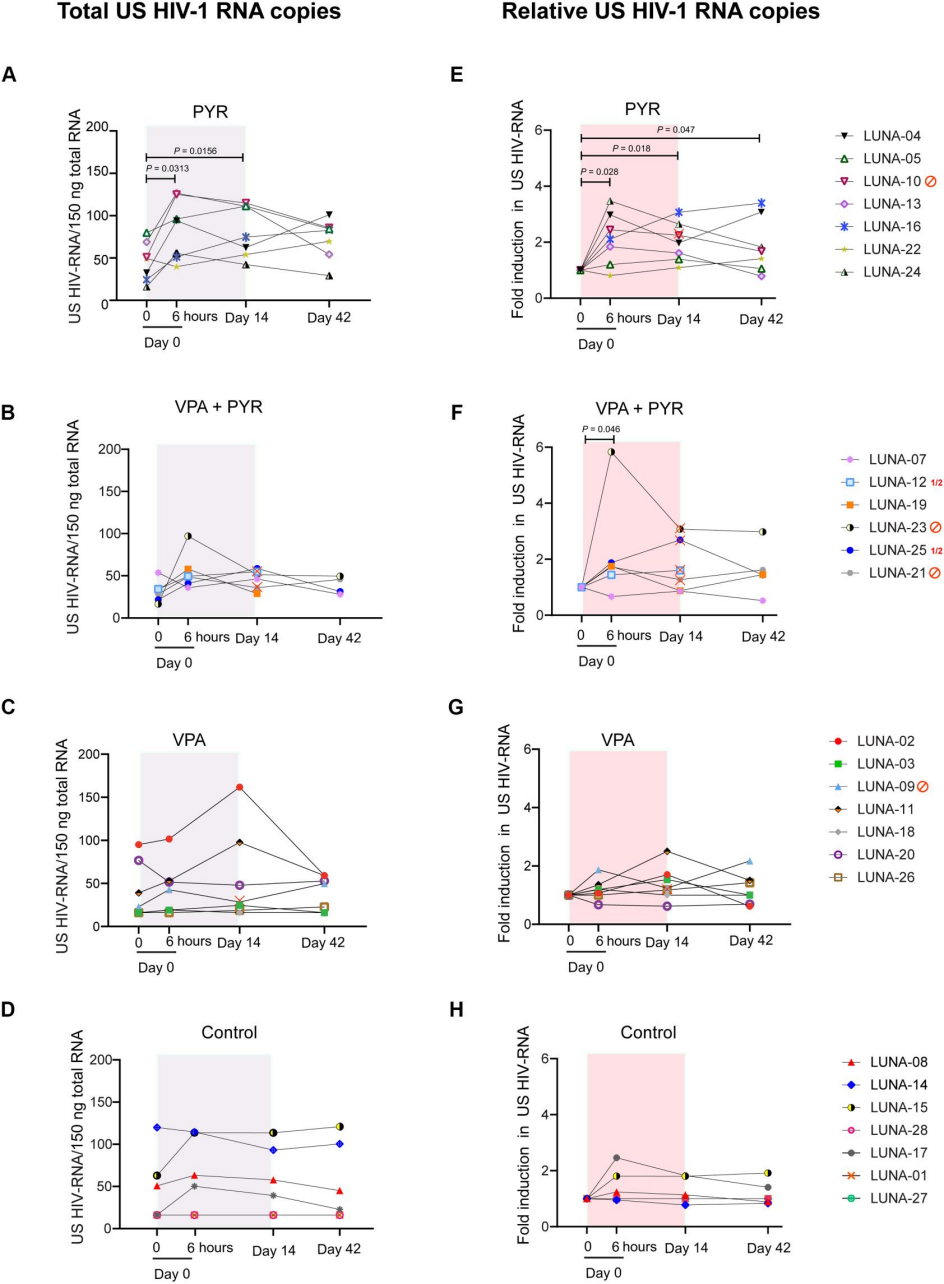
Changes in CA HIV-1 US RNA per participant per arm at four time points during the LUNA study. (**A** to **D**) Graphs represent absolute CA US HIV-1 RNA copies in all participants from the pyrimethamine (A), pyrimethamine with VPA (B), VPA (C), and control (D) arms at treatment initiation (day 0, *t* = 0 hours) after 6 hours after the first dosing (day 0, *t* = 6 hours), at the end of treatment period (day 14), and 28 days after the end of treatment (day 42). (**E** to **H**) Graphs represent fold induction of CA US HIV-1 RNA at different study time points compared to baseline (day 0, *t* = 0) in all participants from the pyrimethamine (E), pyrimethamine with VPA (F), VPA (G), and control (H) arms at the same time points. A total of 1.5 million CD4^+^ T cells were isolated from peripheral blood mononuclear cells, lysed in triplicate, followed by total RNA isolation and reverse transcription. Total copies of CA US HIV-1 RNA were quantified by nested qPCR. The gray/red boxes represent treatment duration. Participants that stopped study medication or had dose adjustments are indicated with either a red stop sign or red ½ symbol and a red cross on the individual points in the graph. In both the VPA arm and the pyrimethamine arm, one participant stopped their study medication on day 8 (LUNA-09) and day 6 (LUNA-10), respectively. In the combination treatment arm, three participants stopped both compounds (LUNA-23 on day 3, LUNA-06 on day 7, and LUNA-21 on day 10), and two participants had their dosage adjusted (LUNA-25 had VPA dose halved from day 2 on, pyrimethamine dose halved from day 7 on, and LUNA-12 had pyrimethamine dose halved from day 7 on). *P* values below 0.05 are indicated. Statistical significance was calculated using Wilcoxon signed-rank tests.

As a consequence of the treatment adjustments, the 6-hour time point after the first dosing is the only sampling time point where all participants were similarly exposed to their allocated therapies and at the same doses. We therefore focused on the effects observed here as the most unbiased measurement time point consequent to drug treatment. From baseline to 6 hours, CA US HIV-1 RNA increased median 2.1-fold (IQR, 1.5 to 2.7; *P* = 0.028) in response to pyrimethamine alone (Fig. 3, A and E) and median 1.7-fold (IQR, 1.4 to 1.9; *P* = 0.046) in response to the combination therapy (Fig. 3, B and F). In contrast, the CA US HIV-1 RNA at this time point from baseline did not change significantly for PLWH allocated to the VPA arm (median fold change, 1.1; IQR, 0.9 to 1.3; *P* = 0.46) (Fig. 3, C and G) or the control arm (median fold change, 1.0; IQR, 1.0 to 1.5; *P* = 0.14; Fig. 3, D and H). Overall, PLWH in the two arms exposed to pyrimethamine had a greater fold change CA US HIV-1 RNA after 6 hours (median, 1.8; IQR, 1.4 to 2.4) compared to those in the two arms without pyrimethamine exposure (median, 1.0; IQR, 1.0 to 1.4; *P* = 0.029).

On pyrimethamine monotherapy, six of seven PLWH responded after the first dose at the 6-hour time point (Fig. 3, A and E). Of these six, five retained an increased CA US HIV-1 RNA during pyrimethamine treatment including LUNA-24 who had CA US HIV-1 RNA below the level of quantitation at baseline. The median fold change from baseline at day 14 was 1.9 (IQR, 1.5 to 2.4; *P* = 0.018), which was in line with the median fold change at 6 hours after dosing (*P* = 0.612) and remained comparable (*P* = 0.50) from days 14 to 42 when a median 1.7 fold change (IQR, 1.2 to 2.5) was still observed. Excluding the single participant (LUNA-10) who stopped pyrimethamine from these analyses did not relevantly alter these median fold change estimates from baseline at day 14 (1.8; IQR, 1.4 to 2.6) or day 42 (1.6; IQR, 1.1 to 3.1). Controls did not exhibit relevant median fold change increases in CA US HIV-1 RNA from baseline at day 14 (1.0; IQR, 1.0 to 1.5; *P* = 0.27) or day 42 (1.0; IQR, 0.9 to 1.2; *P* = 0.47). Two PLWH in the control arm showed some reactivation without a clear clinical explanation, which skewed the estimated effect ranges upward. On VPA, PLWH did not have significantly increased median CA US HIV-1 RNA at any time point compared to pretreatment levels (Fig. 3, C and G) and these responses were also similar to the responses measured in untreated controls (*P* > 0.5 for all comparisons; Table 2). Together, these observations strongly indicate that pyrimethamine induces HIV-1 transcription as early as 6 hours after intake.

We did not observe plasma viremia despite the observed increases in CA US HIV-1 RNA. We found no evidence that pyrimethamine induced plasma viremia at the day 1 time point when all PLWH were on their allocated treatments; five PLWH exposed to pyrimethamine and four PLWH not exposed to pyrimethamine had plasma HIV-1 RNA detected below the assay quantitation limit. These included six PLWH, equally divided by pyrimethamine exposure or not, who had no viral genome detected before drug administration. When evaluating the entire 6-week trial period, plasma HIV-1 RNA remained below the lower limit of quantification (LOQ) in the majority of participants (table S6) except for two participants on VPA, one participant on pyrimethamine, and one participant on combination treatment (all <50 copies/ml). These findings indicate that the intracellular effect of pyrimethamine did not result in measurable cell-free HIV-1 RNA in the plasma of participants.

### Pyrimethamine and VPA do not synergize to induce HIV-1 transcription in vivo

Because of the treatment adjustments that occurred in the combination treatment arm, we used the measurements at 6 hours after the first dose to test the hypothesis that synergy occurred between pyrimethamine and VPA in the context of their combined latency reversal activity on HIV-1. In PLWH exposed to the combinatorial treatment, five of six PLWH had increased CA US HIV-1 RNA 6 hours after the first dose (Fig. 3, B and F). There was one individual (LUNA-23) with a particularly large 5.83-fold increase, while the other five PLWH had fold changes ranging from 0.67 to 1.88. Apart from an HIV-1 CRF02-AG subtype infection, the clinical characteristics of this participant were fitting the characteristics of the overall study population. At this 6-hour time point, the median fold change response in the combination treatment arm did not differ significantly from the median response observed with pyrimethamine alone (*P* = 0.475). As per protocol defined, the data also formally excluded synergy since the mean fold change and its full 95% confidence interval (2.2, 0.3 to 4.1) did not exceed the expected mean fold change of 2.25, which was based on the mean fold change effects estimated in the pyrimethamine (2.11) and VPA (1.14) arms separately. Hence, synergy could not be demonstrated and neither do these data indicate additive effects.

In the four PLWH (LUNA-07, LUNA-12, LUNA-19, and LUNA-25) in the combination arm who continued uninterrupted pyrimethamine exposure throughout the study, the median fold change in CA US HIV-1 RNA was 1.2 (IQR, 0.9 to 2.1) at day 14. This effect was comparable to the changes observed when evaluating all PLWH including those who stopped combinatorial treatment (median, 1.4; IQR, 0.9 to 2.7). Unfortunately, the CD4^+^ T cells yield from the samples were insufficient for LUNA-12 and LUNA-19 to measure CA US HIV-1 RNA at the day 42 end point. Given the treatment adjustment in LUNA-25, at this time point, only LUNA-07 had completed the full allocated treatment and had end point results available. This participant did not exhibit CA US HIV-1 RNA increases at any time point. Apart from a longer duration of plasma viral suppression in the upper quartile of the cohort (nearly 12 years), the other baseline characteristics in this participant were consistent with the general cohort. When evaluating the four PLWH who had exposure to pyrimethamine in this combination treatment arm and with samples at day 42 available (LUNA-07, LUNA-21, LUNA-23, and LUNA-25), the median fold change was 1.5 (IQR, 1.0 to 2.3), comparable to pyrimethamine alone and not significantly different from controls (Table 2). Overall, these data support the conclusion that valproic did not augment the activity of pyrimethamine in reactivating HIV-1.

### Lower pyrimethamine plasma levels occur with concomitant VPA exposure

**Fig. 4.**
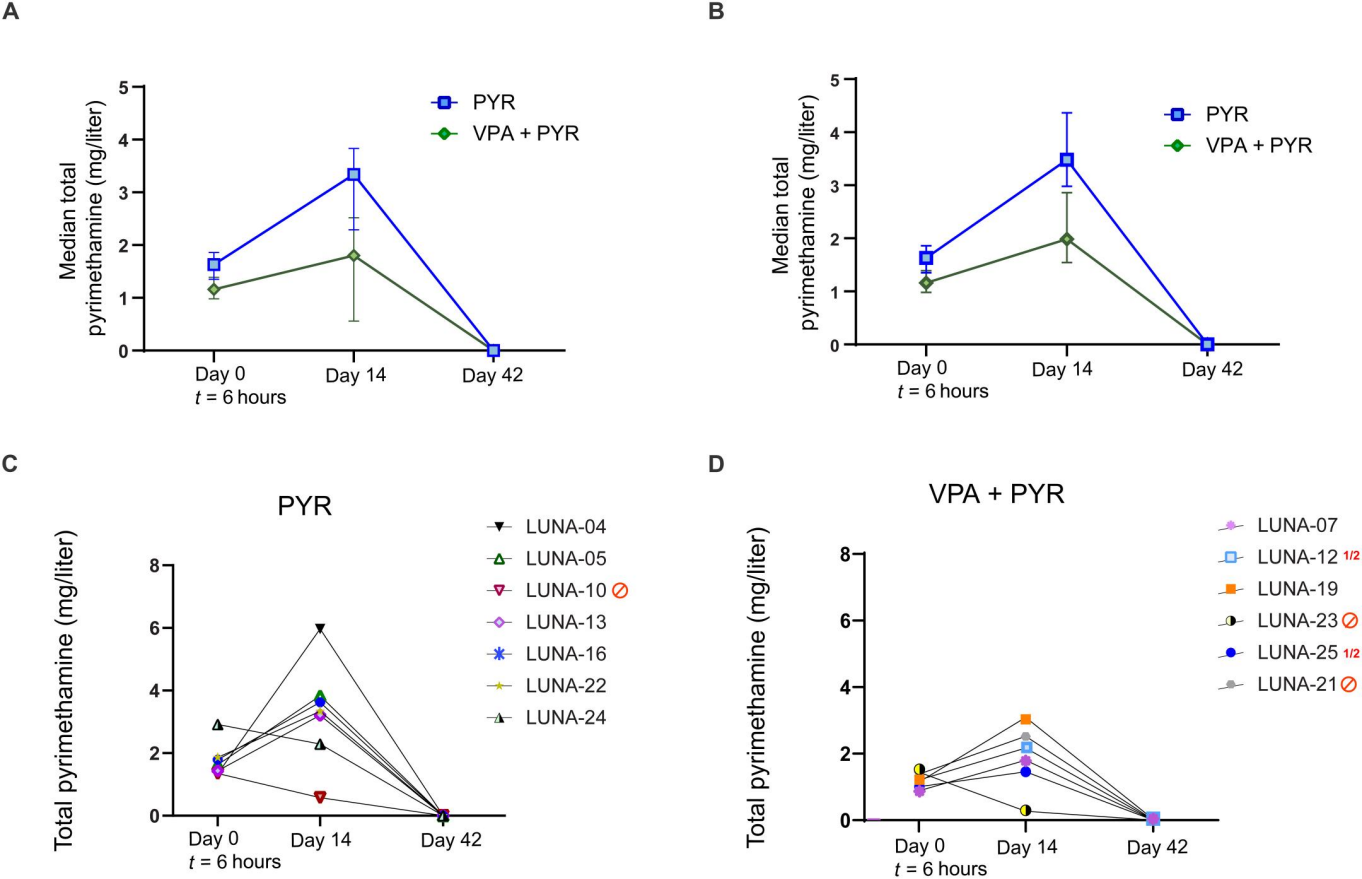
Pharmacokinetics of the drug pyrimethamine in study participants. (**A** and **B**) Pharmacokinetic analysis of the drug pyrimethamine in study participants from pyrimethamine (blue) and the combination treatment arm with pyrimethamine and VPA (green) as measured by median total pyrimethamine (mg/liter) levels in plasma with IQRs at 6 hours after first dosing (day 0, *t* = 6 hours), at the end of treatment period (day 14), and 28 days after the end of treatment (day 42) overall (A) and in those with uninterrupted pyrimethamine exposure (B) and represented as plasma pyrimethamine levels at the time points per individual on pyrimethamine (**C**) or the combination treatment with pyrimethamine and VPA (**D**).

For this trial, we established an in-house assay to measure total pyrimethamine plasma levels (Fig. 4). At the 6-hour time point, the median plasma total pyrimethamine concentration in the seven participants that received solely pyrimethamine was 1.63 mg/liter (IQR, 1.40 to 1.83), and in the combination arm, this was 1.16 mg/liter (IQR, 1.0 to 1.31) (Fig. 4A). This difference was more accentuated at day 14 when the six PLWH in the monotherapy arm and the four PLWH in the combination therapy arm with uninterrupted pyrimethamine exposure had median *C*_trough_ of 3.48 mg/liter (IQR, 3.21 to 3.83) and 1.99 mg/liter (1.63 to 2.63) (Fig. 4B). Although these concentrations fall within the expected range from previous human studies (*49*–*52*, *60*), and despite taking out those who stopped pyrimethamine before day 14 from the analysis, the data suggest lower pyrimethamine concentrations when combined with VPA (Fig. 4). This might be the result of protein binding displacement or an unexpected drug-drug interaction. We did not find such an effect when comparing the median plasma total VPA concentration at the 6-hour time point between monotherapy (median, 47.3 mg/liter; IQR, 47.2 to 60.1) and combination treatment (47.4 mg/liter; IQR, 26.0 to 57.3) (fig. S2). The median *C*_trough_ observed at the day 14 time point in the six PLWH with uninterrupted exposure to VPA monotherapy and the four PLWH with uninterrupted exposure to VPA in the combination treatment was 84.8 mg/liter (36.4 to 99.6) and 113.2 mg/liter (IQR, 83.1 to 137.5), respectively, both above VPA’s therapeutic range lower border for epilepsy (50 mg/liter). We found no correlations between pyrimethamine plasma exposure and the fold change in CA US HIV-1 RNA from baseline at 6 hours (*r* = 0.26, *P* = 0.39) or day 14 (*r* = 0.041, *P* = 0.91) in those exposed to pyrimethamine or in the participants from the two pyrimethamine arms separately (fig. S3). We explored median pyrimethamine *C*_trough_ by integrase inhibitor exposure but found no relevant differences at 6 hours (1.3 mg/liter versus 1.4 mg/liter) or day 14 (2.8 mg/liter versus 3.2 mg/liter) in those with uninterrupted pyrimethamine. Conversely, median unbound dolutegravir concentrations were comparable between those with and without pyrimethamine exposure at 6 hours (11.7 μg/liter versus 10.1 μg/liter) but decreased during the treatment course. This effect has been attributed to VPA by protein displacement as we described previously (*61*). PLWH with exposure to efavirenz- or nevirapine-containing cART (both CYP3A inducers) did not have lower median plasma concentration of pyrimethamine (a CYP3A substrate). No pyrimethamine or VPA was detectable in the plasma at day 42. The combined data do not support relevant drug-drug interactions between pyrimethamine and dolutegravir but do support relevant drug-drug interactions of VPA on dolutegravir and pyrimethamine.

We found no clear outliers in the data on pyrimethamine and VPA plasma exposure for the seven PLWH who had treatment adjustments for toxic effects (table S6). Of those seven, the ones in the pyrimethamine-containing arms had pyrimethamine plasma levels at 6 hours after the first dose that were at or below the median plasma exposure found in the pyrimethamine monotherapy arm at that time points. All participants where pyrimethamine was stopped had clearly lower plasma levels at day 14 with the notable exception of LUNA-21 (2.52 mg/liter) who stopped treatment at day10, later than the others who interrupted pyrimethamine (table S6). In the same line, three of the five PLWH, who had their therapy adjusted for toxicity while receiving VPA, had plasma levels at day 7 exceeding the therapeutic window defined for treating epilepsy (up to 100 mg/liter) compared to three of nine who continued VPA (table S6). This provides some evidence that VPA plasma exposure related to clinical toxicity, comparable to the use of therapeutic drug monitoring of VPA for other conditions (*62*). For pyrimethamine, the therapeutic window and relationship between plasma exposure and toxicity are far less well defined but no exceptional high plasma exposures were found.

### Pyrimethamine induces expression of biomarkers associated with BAF inhibition

**Fig. 5.**
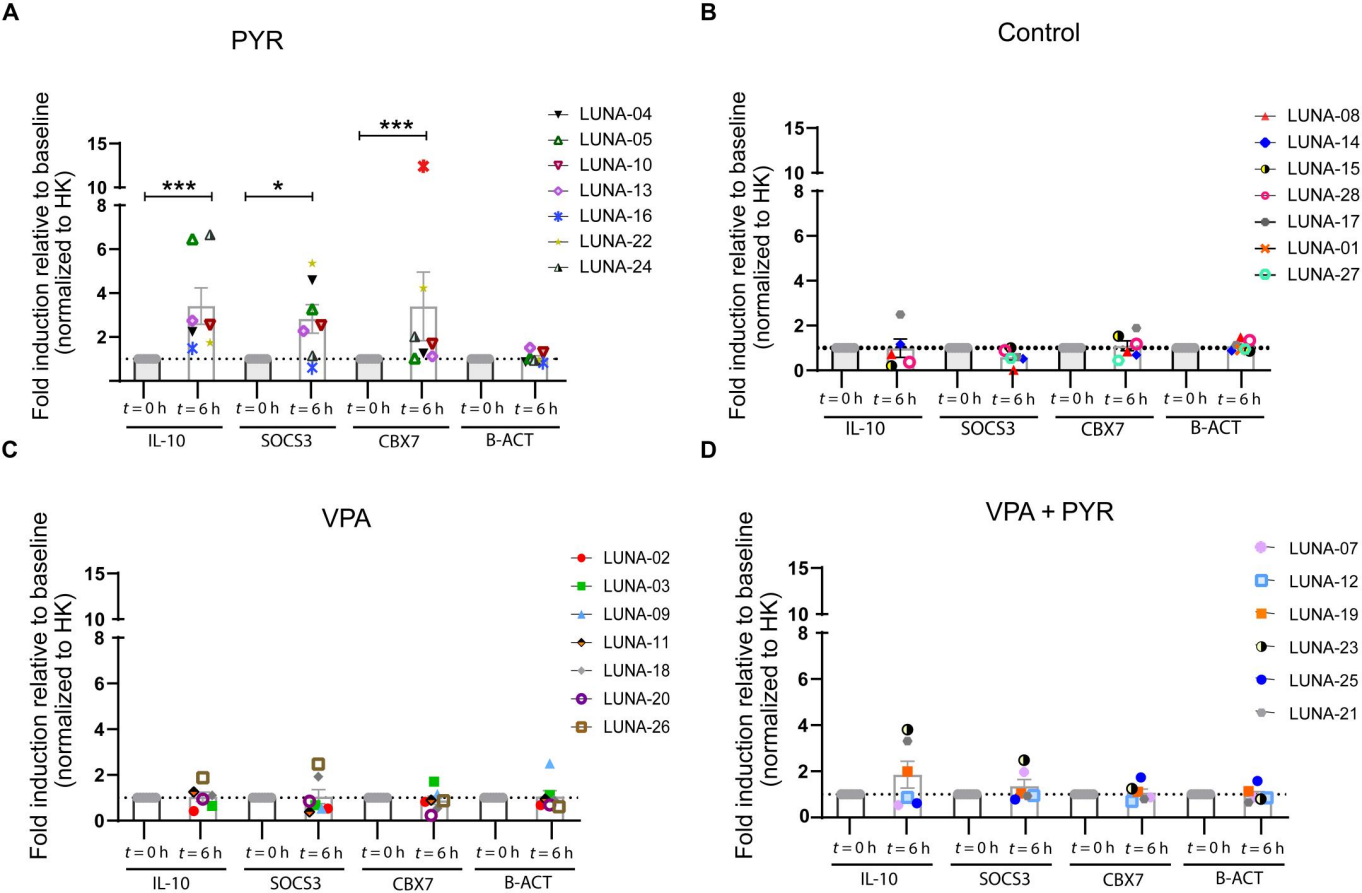
Pharmacodynamics of the drug pyrimethamine in study participants. (**A** to **D**) Gene expression profile of BAF complex molecular targets IL-10, SOCS3, and CBX7 in all participants from pyrimethamine (A), control (B), VPA (C), and the combination treatment arm with pyrimethamine and VPA (D) at 6 hours after first dosing (day 0, *t* = 6 hours). Graphs represent mean (SEM) fold induction relative to pretreatment level (day 0, *t* = 0 hours). Cyclophilin A was used as a housekeeping (HK) gene for normalization, and β-actin (B-ACT) was used as a control. *P* values were calculated using Mann-Whitney *U* test with * representing *P* < 0.05 and *** representing *P* < 0.01.

Pharmacological inhibition of the BAF complex by pyrimethamine leads to functional changes in the expression levels of several genes (*44*, *63*–*66*). To assess the pharmacodynamics, activity, and specificity of pyrimethamine in this clinical trial, we analyzed the gene expression profile of three target genes of the BAF complex (*IL-10*, *SOCS3*, and *CBX7*) and a control gene (*B-ACT*) on day 0 before treatment and day 0 at 6 hours after the first dose. We reasoned that the effect on the expression of these gene targets at later time points (days 14 and 42) would be less useful as specific biomarker of BAF complex inhibition due to treatment interruptions and potential induction via other pathways. We observed a significant fold increase in the median gene expression levels of *IL-10*, *SOCS3*, and *CBX7* (2.55-, 2.53-, and 1.68-fold, respectively; table S7) in the pyrimethamine treatment arm (Fig. 5A). As expected, we observed no increase in the mRNA levels of these genes in either the control arm or the VPA intervention arm (Fig. 5, B and C), indicating that these molecular targets are specific for pyrimethamine. No significant increase in gene expression levels of BAF target genes was observed in the combined intervention arm (Fig. 5D). These findings are consistent with, and might be the consequence of, the generally lower plasma pyrimethamine *C*_trough_ in participants that received the combined intervention regimen compared to those that received solely pyrimethamine.

### Pyrimethamine exposure does not lead to a reduction of the inducible HIV-1 reservoir

**Fig. 6.**
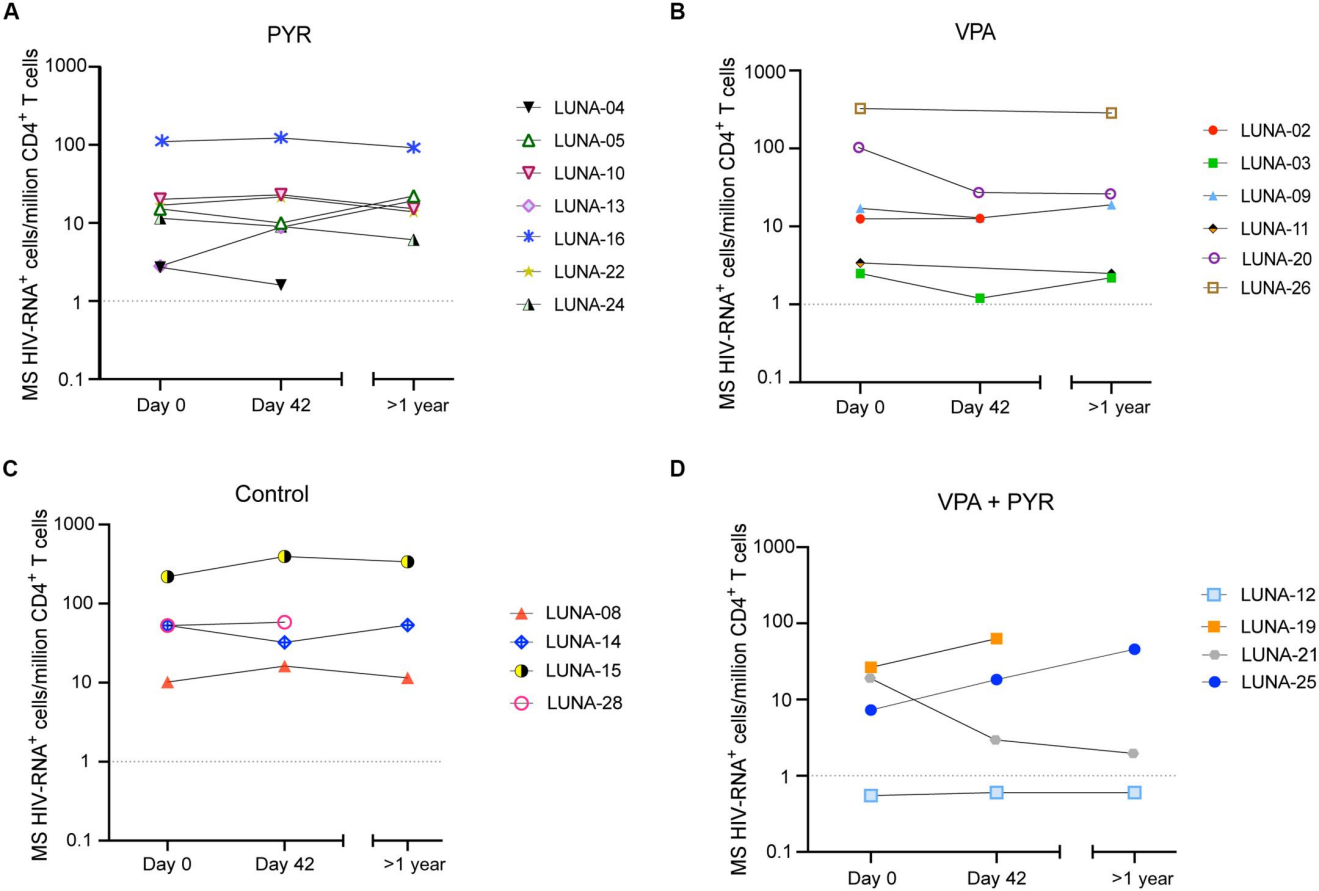
Effect of the intervention regimen on the HIV-1 inducible reservoir size. (**A** to **D**) Graphs represent measurements of inducible reservoir size in samples obtained from participants from pyrimethamine (A), VPA (B), control (C), and the combination treatment arm with pyrimethamine and VPA (D) at treatment initiation (day 0, *t* = 0 hours), 28 days after the end of treatment (day 42), and >1 year after the end of treatment. Isolated CD4^+^ T cells were activated ex vivo with PMA/ionomycin for 12 hours, and the frequency of CD4^+^ T cells expressing MS HIV-1 RNA was determined using a TILDA. The dotted horizontal line represents the assay limit of detection.

To assess whether in vivo HIV-1 transcription induced by pyrimethamine or VPA alone or in combination affected the inducible viral reservoir, we used a tat/rev-induced limiting dilution assay (TILDA) to quantify the frequency of cells expressing multiply spliced (MS) HIV-1 RNA upon ex vivo cellular activation with phorbol 12-myristate 13-acetate (PMA)/ionomycin at baseline (day 0, *t* = 0 hours) and day 42 (Fig. 6 and fig. S4, A to C) (*67*, *68*). Since low cell yields at day 42 occurred in several individuals during the trial, which would impede accurate reservoir assessments, we decided during the trial, before data collection and analysis, to invite all participants back when all were more than 1 year after day 42. A total of 27 of 28 PLWH responded and were sampled at a 1- to 2.2-year range after their last visit (table S8). Ultimately, TILDA could be performed for 20 to 28 study participants with available samples (71%) including all participants on pyrimethamine monotherapy. For eight participants without TILDA data, sample material was insufficient in four (three on combination treatment and one control) and four had non-B HIV-1 subtypes (including one participant in the combination arm). For the remaining 10 participants with pyrimethamine exposure that could be evaluated, 2 on pyrimethamine alone (LUNA-05 and LUNA-22) had insufficient cells available at day 42. Here, the measurement from the cells obtained >1 year after the last visit was used.

The overall results do not indicate a significant change in the reservoir size with pyrimethamine exposure (Fig. 6A). The reservoir was estimated at median 15.1 (range, 2.7 to 109.8) cells per million CD4^+^ T cells before treatment with pyrimethamine alone and at 10.0 (range, 1.6 to 122.8) cells per million CD4^+^ T cells after treatment. This variation was well within the assay coefficient of variation (25.1%). The reservoir remained stable on VPA: median 14.8 cells per million CD4^+^ T cells at day 0 and median 19.4 CD4^+^ T cells per million at day 42 (Fig. 6B). The number of participants with reservoir results available in the other two arms (*n* = 3 in each) was considerably lower and precluded statistical inferences, but the data points that are available do not challenge the main conclusion that overall no decrease in the reservoir can be observed (Fig. 6, C and D). In one of these participants (LUNA-12, combination arm), TILDA was below the limit of detection (LOD) at all sampling points (Fig. 6D). Individual level changes in reservoir size were observed in a post hoc analysis of an at least twofold increase or decrease in four (LUNA-13, LUNA-15, LUNA-19, and LUNA-25) and three participants (LUNA-03, LUNA-20, and LUNA-21), respectively, between days 0 and 42 (Fig. 6) without signs of clustering to a specific treatment arm or an associated clinical predictor variable.

## DISCUSSION

The BAF complex is an important molecular regulator of HIV-1 latency and offers a new yet clinically unstudied pharmacological target for latency reversal. In this randomized controlled trial, we demonstrated that BAF complex inhibition by pyrimethamine induces cellular HIV-1 transcription and reversal of latency in cART-suppressed PLWH. A comparable effect on the reservoir by pyrimethamine was also observed when VPA was added as partner LRA. Thus, the combination treatment failed to show synergistic or additive effects. Furthermore, interventional treatment adjustments occurred most frequently in PLWH randomized to the combinatorial arm, although the number and grade of the reported AE were in line with the ones observed in the individual monotherapy arms. We cannot exclude the possibility of physician- or participant-directed biases in the combinatorial arm where both the treating physician and the participant were aware of the higher drug burden. However, we believe that this trial, as the first randomized controlled clinical study on combinations of LRA, underlines that testing combinatorial LRA approaches is feasible. It also highlights the need to monitor for drug tolerability and provides a safety benchmark for future studies combining LRAs in the clinical HIV-1 cure trial field.

The most important finding is the identification of pyrimethamine as an effective drug to reverse HIV-1 latency in PLWH. It acts through a novel mechanism of action that we unraveled first in in vitro and ex vivo studies and now successfully moved into clinical practice (*44*, *48*). We found an approximately twofold induction of cellular HIV-1 transcription from pretreatment levels that persisted during treatment. The effect on the proviral reservoir was only observed in the presence of measurable pyrimethamine plasma concentrations, accompanied by selective induction of other BAF complex target genes and not observed in individuals that were not exposed to pyrimethamine. These observations support that the inhibition of the BAF complex at the HIV-1 promotor region is responsible for this effect in vivo, comparable to its established working mechanism in vitro. In addition, although BAF complex inhibition by pyrimethamine was able to induce HIV-1 transcription in vivo, it did not lead to a reduction in the inducible reservoir. This might be due to an insufficient antigen production and further recognition by cytotoxic immune cells. Of note, a total of four participants had an apparent increase in the size of the inducible reservoir in a post hoc analysis. We did not find a defining clinical characteristic or common factor underlying these observations. Because some fluctuation was also observed in the control arm, clustering to any arm was absent, it is currently unclear whether these observations on an individual level are reflective of, for example, the antiretroviral or LRA treatment course in specific participants or are rather due to limitations of viral reservoir quantification tools or natural variability. Future studies should take these possibilities into consideration.

With regard to VPA, we optimized the dosing and used an acid-resistant formulation for a more direct gastrointestinal absorption unlike the previous studies where underdosing or underexposure might have been the cause of the observed discrepant effects of VPA as LRA (*17*–*22*). In our trial, participants receiving VPA had higher plasma levels than those reported in prior studies (*17*–*22*). Although higher systemic VPA peak concentrations could more likely induce HIV-1 reactivation, we did not observe an increase in CA US HIV-1 RNA or viral plasma load in PLWH treated with VPA alone.

Strategies that use combinatorial approaches of LRAs could result in higher efficacy in reactivating HIV-1 latency (*5*). We have shown in previous studies that the effect of pyrimethamine on HIV-1 latency ex vivo could be potentiated when combined with other LRAs including HDACi (*44*, *46*). We therefore designed the study to include a combinatorial arm to test whether the effect of the individual LRAs could be potentiated in vivo. Our results showed, however, no synergistic or additive effects on the HIV-1 reservoir in PLWH in the combinatorial arm. Future HIV-1 cure studies using pyrimethamine would benefit from combinations with other LRAs that could induce higher levels of HIV-1 transcription and compounds that enhance immune-mediated killing.

To put our clinical study and the role of pyrimethamine as an LRA in the context of HIV-1 cure clinical studies, we compared the effect of pyrimethamine on HIV-1 latency with other LRAs tested in PLWH. First, the effect of pyrimethamine on HIV-1 transcription did not appear to augment with multiple dosing. This is in contrast to observations made in studies with other LRAs from the HDACi class and disulfiram, where the mean effect on cellular HIV-1 transcription increased with sequential dosing during treatment (*26*, *34*, *39*, *40*). Our observation fits a more dichotomous (on/off) effect dependent on whether sufficient intracellular concentrations are reached rather than a gradual effect seen with HDACi, where multiple doses are apparently necessary to reach a maximum effect. Our prior in vitro observations showing that the displacement of the BAF complex at the nucleosome 1 region of the HIV-1 promotor locus was comparable at different dosages support this assumption (*44*). A certain maximized potential to reactivate HIV-1 is also suggested since a further increase in pyrimethamine plasma levels did not result in an additional CA US HIV-1 RNA increase during the trial. Second, pyrimethamine’s effect waned several weeks after the last drug exposure. This is in line with most other trials on effective LRAs that act through derepressing HIV-1 transcription (*34*, *35*, *39*) with the exception of one trial on vorinostat (*26*). Third, the combinatorial approach used in this trial with pyrimethamine and a derepressor HDACi as a partner LRA may be less likely to work synergistically compared to combining a derepressing and an activating LRA. Our data support this hypothesis, although we certainly acknowledge that different results may have been obtained with a more potent partnering HDACi. Last, regarding the observed safety profile, pyrimethamine as monotherapy had a favorable clinical safety profile, comparable to studied effective HDACi in HIV, with only mild AE observed. Pyrimethamine’s additional advantages of being a globally available, orally administered, inexpensive drug together with the considerable experience to treat AIDS patients with it further support its use in follow-up cure studies. These future studies should nonetheless focus on optimizing the necessary pyrimethamine dose to reactivate HIV-1 latency while minimizing drug exposure and toxicity, identifying the relevance of the used loading dose and treatment duration, and, given our trial findings, determine the optimal partner drug for pyrimethamine in terms of efficacy and safety.

In our study, pyrimethamine alone led to a twofold increase in levels of CA US HIV-1 RNA at the time point where all participants had received their allocated dose. While this effect is rather modest, it is comparable to recent reports using other classes of LRAs. The HDACi VPA did not result in an increase in CA US HIV-1 RNA in this trial, as opposed to what has been observed with other HDACi such as vorinostat, romidepsin, and panobinostat (*26*, *28*, *33*–*39*). In a study more comparably designed to our study, CA US HIV-1 RNA increased a mean 2.6-fold at time points during vorinostat (*26*). No reactivation was, however, observed when vorinostat was combined with a T cell–inducing HIV-1 vaccine in PLWH who initiated cART during acute HIV-1 (*28*), and a 1.5 fold change in CA US HIV-1 RNA was observed with vorinostat treatment in a recent study in postmenopausal women (*33*). For the HDACi romidepsin, initial studies reported an approximately threefold increase in CA US HIV-1 RNA (*35*, *36*). Additional studies on romidepsin’s pharmacodynamics profile unexpectedly found no significant effect on HIV-1 transcription (*38*). In trials that combined romidepsin with 3BNC117 or a therapeutic HIV-1 vaccine, cellular HIV-1 transcript levels generally changed overall less than threefold (*37*, *39*). This was also true for the reactivation effects observed with disulfiram, bryostatin, a Toll-like receptor 9 (TLR9) agonist, or pembrolizumab (*40*–*43*). Another HDACi, panobinostat, reactivated cellular HIV-1 transcription more potent compared to all other HDACis in the only clinical trial with this compound (*34*). Overall, when comparing pyrimethamine to other LRAs, its effect to reactivate HIV-1 transcription can be classified as at least comparable.

The assay we developed and validated to measure pyrimethamine plasma levels turned out to be critical since, unexpectedly, the combination arm had lower pyrimethamine plasma levels with a concomitant lower induction of various BAF target genes. Given that both pyrimethamine and VPA are highly protein-bound, a plausible explanation for this effect could be attributed to plasma protein-protein competition dynamics, although further research needs to be conducted to support this statement. We identified a similar mechanism previously where VPA affected dolutegravir levels (*61*). This signals that routine pharmacokinetic profiling of the interventional drugs and cART should be included in the design of future cure trials, even if no interaction is expected. This is particularly important when testing combinations of LRAs.

This proof-of-concept study has strengths and limitations. The study’s main strength is the inclusion of a control arm and a combination arm that allowed us to assess the specific effect of pyrimethamine alone and in combination with an HDACi. Regarding limitations, although not exceeding predefined safety criteria, the combinatorial arm faced a disproportionate amount of treatment adjustments before the end of the intervention period. This influenced our ability to draw solid conclusions on potential late LRA effects combination therapy might have. However, the absence of an effect with VPA alone together with the absent synergism or additive effects in PLWH on the combination of drugs makes potential late effects less likely. Our sampling strategy for this pilot favored having many time points for the primary end point over blood quantity for in-depth analyses. Leukapheresis was practically not possible. This turned out to be a challenging strategy in some cases, especially in individuals with less blood CD4^+^ T cells. We prioritized using CD4^+^ T cells for the main end points, although this meant that in-depth analysis of resting memory CD4^+^ T cells or other cell types was therefore not possible. Another challenge was to measure the size of the reservoir by TILDA posttreatment at day 42 as a main secondary end point. This was not possible in four PLWH due to limited sample availability and four PLWH had non–B subtypes that resulted in critical primer-probe mismatches. However, all PLWH in the most relevant arm with pyrimethamine had a sufficient amount of cells for reservoir analysis. We amended the protocol and invited all participants for an additional sampling at least 1 year after the last study visit with the purpose to more confidently support the reservoir findings. We did not observe marked changes in the size of the latent reservoir over this time course. As a pilot study, the exploratory analyses and relatively small sample size could have resulted in type 1 and 2 errors, respectively. Future LRA trial should take into account these limitations for initial design with regard to diversity in participant inclusion, assays, sampling time points, and materials required.

In summary, inhibition of the BAF complex by pyrimethamine resulted in modest but significant reversal of HIV-1 latency in PLWH immediately after the first dose and persisted during treatment course. Our data are supportive of the BAF inhibitor pyrimethamine as a novel drug option in the LRA arsenal, which has widespread use in the clinic, excellent safety profile, and favorable pharmacological features, including its excellent brain penetration that offers potential for penetration in HIV-1 reservoir sanctuary sites (*50*, *53*, *54*, *56*, *60*). Thus, pyrimethamine is an attractive candidate for inclusion in future pharmacological approaches toward an HIV-1 cure.

## MATERIALS AND METHODS

### Eligibility

The LUNA trial (clinicaltrials.gov identifier: NCT03525730) is a proof-of-concept, four-arm, open-label randomized controlled interventional clinical trial to assess the effect of the BAF inhibitor pyrimethamine on the HIV-1 reservoir when given alone or as a combination of LRAs with the HDACi VPA. Individuals aged ≥18 years visiting the outpatient department of infectious diseases at the Erasmus University Medical Center (Rotterdam, the Netherlands) were eligible if they had a confirmed HIV-1 infection and a CD4^+^ T cell count of at least 200 cells/μl, receiving cART with a pre-cART HIV-1 RNA zenith of ≥10,000 copies/ml and plasma HIV-1 RNA levels of <20 copies/ml in the year before the intervention (with a minimum of two measurements taken). Exclusion criteria were female with a reproductive potential due to VPA’s teratogenic potential, previous virological failure with resistance-associated mutations acquired on cART, active hepatitis B or C infection, prior exposure to LRAs, immunomodulating medication, or medication known to interact with VPA or pyrimethamine. The inclusion period of the study was between April 2018 and September 2020 following approval by the Medical Research Ethics Committee (MEC-2017-476) in accordance with the principles of the Helsinki Declaration. The protocol was amended once in February 2020 before the analysis of end points. The reason for this amendment was to allow additional blood sampling to overcome potential low cell yields for a main end point analysis on the reservoir size. All participants provided written informed consent. The full protocol is available as appendix, and a synopsis is available in the Supplementary Materials. Study reporting follows the CONSORT reporting guidelines.

### Intervention

Eligible and consenting trial participants were screened within 6 weeks before the start of the intervention phase and subsequently randomized to one of four arms to receive oral doses of either: pyrimethamine once a day for 14 days (200 mg on the first day followed by 100 mg onward), VPA (30 mg/kg per day) (divided into two equal doses) for 14 days, a combination of pyrimethamine and VPA dosed likewise, or no intervention. For the randomization process, an independent statistician delivered sealed opaque envelopes that included the study identifier with the allocated treatment based on random allocation performed in R. VPA and pyrimethamine were administered in dosages used in the chronic treatment of epilepsy (*62*), and previous clinical studies on the treatment of cerebral toxoplasmosis in AIDS patients or as prophylaxis or malarial treatment in pregnant women (*49*, *53*–*57*). The dose and duration of VPA and pyrimethamine treatment, and the timing of viral reservoir analysis on day 0 after 6 hours, was based on *T*_max_ of both compounds, available literature, expert opinion, and our previous work (*69*–*71*). Blood samples were collected 6 hours after the participants received the intervention regimen assuming *T*_max_ to be reached by pyrimethamine and VPA after approximately 4 hours, and an additional 2 to 3 hours for HIV-1 transcription and HIV-1 RNA accumulation (*24*). To achieve a constant and the earliest *T*_max_ possible, participants received their first dose of the intervention regimen on day 0 in a fasted state. On day 0, cART and study medication intake were taken under direct supervision of the study staff. The 2-week intervention phase was followed by a 4-week post-intervention phase. Overall, participation involved 11 study visits. To obtain an additional post-intervention phase measurement for the inducible HIV-1 reservoir size, the study was amended and participants were asked to give written consent to an extra blood draw after the 4-week post-intervention phase ended. Plasma HIV-1 RNA levels (COBAS TaqMan; Roche, Basel, Switzerland; LOQ, 20 copies/ml) were monitored at all 11 visits. CD4^+^ T cell counts were determined at screening, at the start and end of the intervention phase (days 0 and 14), at day 42, and at the post-intervention phase (≥day 42).

### Safety

Hematological parameters and kidney and liver function were monitored before treatment and on days 7, 14, and 42. At each study visit, participants were clinically assessed by the study physician and safety assessments were performed. All participant-reported AE and SAE were evaluated in relation to VPA and pyrimethamine. Severity was graded according to the Common Terminology Criteria for Adverse Events (CTCAE) version 4.0. In the study protocol, a number of stopping rules were defined including a prespecified interim analysis after the inclusion of 14 participants focusing on the number of participants discontinuing study medication during the intervention phase, CD4^+^ T cell count <200 cells/μl, possibly drug-related SAE/AE ≥ grade 4, or AIDS-related illness (Centers for Disease Control category C events).

### Study end points

The prespecified primary end point was the change in HIV-1 reactivation at treatment initiation and at the end of treatment in the study arms, measured as the change in CA US HIV-1 RNA between treatment initiation (day 0 at 0 hours) and at the end of the study (day 42). Secondary end points reported here included the change in inducible HIV-1 reservoir size as quantified by TILDA, the synergistic effects of pyrimethamine with VPA on the induction of CA US HIV-1 RNA, the change in CA US HIV-1 RNA between time points within and between study arms, plasma HIV-1 RNA changes, the pharmacokinetic and pharmacodynamic profiles of the intervention regimens, and the clinical safety and tolerability of the intervention regimen.

### CA US HIV-1 RNA

For quantification of CA US HIV-1 RNA, CD4^+^ T cells were isolated from peripheral blood mononuclear cells (PBMCs) by negative selection using EasySep Human CD4^+^ T Cell Enrichment Cocktail (STEMCELL Technologies). Isolation of CD4^+^ T cells was performed on ice or at 4°C (unless indicated otherwise by the manufacturer). Approximately 1.5 × 10^6^ CD4^+^ T cells were lysed in triplicate with TRI reagent. Total RNA was isolated by the phenol/chloroform isolation method following the manufacturer’s instructions. A minimum of 150 ng of total RNA was deoxyribonuclease (DNase)–treated following the manufacturer’s instructions (DNase I, Amplification Grade, Invitrogen), and complementary DNA (cDNA) synthesis was performed in duplicate with SuperScript II (Invitrogen) following the manufacturer‘s instructions. Absolute quantification of CA US HIV-1 RNA was performed following a modified version of Pasternak *et al.* methodology (*72*). Briefly, the first round of nested amplification was performed in a final volume of 25 μl using 10 μl of cDNA, 2.5 μl of 10× PCR buffer (Life Technologies), 1 μl of 50 mM MgCl_2_ (Life Technologies), 1 μl of 10 mM deoxynucleotide triphosphate (dNTP) (Life Technologies), 0.075 μl of 100 μM US forward primer, 0.075 μl of 100 μM US reverse 1 primer, and 0.2 μl of Platinum Taq polymerase (Life Technologies) at 95°C for 5 min, followed by 15 cycles at 95°C for 30 s, 55°C for 30 s, and 72°C for 15 min. The second round of amplification was performed in a final volume of 25 μl using 2 μl of preamplified cDNA, 2.5 μl of 1× PCR buffer (Life Technologies), 1 μl of 50 mM MgCl_2_ (Life Technologies), 1 μl of 10 mM dNTPs (Life Technologies), 0.05 μl of 100 μM US forward primer, 0.05 μl of 100 μM US reverse 2 primer, 0.0375 μl of US probe, and 0.2 μl of Platinum Taq polymerase (Life Technologies) at 95°C for 5 min, followed by 45 cycles at 95°C for 30 s and 60°C for 1 min. The list of primers and probe is available in table S9. The absolute number of US copies in the PCR was calculated using a standard curve ranging from 2 to 512 copies of a plasmid containing the full-length HIV-1 genome (pNL4.3.Luc.R-E-). On the basis of standard curve analysis, we assigned a LOQ of 16 copies with an intra-assay coefficient of variation of <5% (fig. S4D). The quantity of CA US HIV-1 RNA was expressed as the number of copies per 150 ng of input RNA in reverse transcription.

### BAF complex target gene expression

RT-qPCR reactions were conducted using GoTaq qPCR Master Mix (Promega) following the manufacturer’s protocol. The following thermal cycling protocol was used for amplification: 3 min at 95°C, followed by 40 cycles of 95°C for 10 s and 60°C for 30 s. Expression data were calculated using the 2^−∆∆Ct^ methodology (*73*). Cyclophilin A was used as a housekeeping gene for the analysis. The list of primers is available in table S9.

### Pharmacokinetic analysis

Self-reported adherence was assessed at each study visit, and empty pill strips were collected after the 2-week intervention phase. EDTA plasma samples were collected at the start of the intervention phase on day 0 (at 0 hours and at 6 hours after the first dose), day 7 (VPA only), day 14, and day 42. Plasma concentrations of VPA were analyzed by using a routinely implemented and validated assay (Multigent Valproic Acid Assays) at the Erasmus University Medical Center Pharmacy. For pyrimethamine measurements, we developed an assay (validated according to U.S. Food and Administration/European Medicines Agency guidelines) using the Waters Acquity UPLC-MS/MS. Samples (50 μl) were prepared and pumped into the column [Waters Acquity BEH C18 (1.7 μm, 2.1 × 100 mm), at 50°C]. A gradient (0.3 ml/min) with two eluents was used (A: 2 mM ammonium acetate + 0.1% formic acid in water; B: 2 mM ammonium acetate + 0.1% formic acid in methanol). The total run time was 4.2 min. Mass transitions used were mass/charge ratio (*m/z*) 249.09 to 176.97 (pyrimethamine) and *m/z* 325.05 to 307.05 (quinine), cone voltages were 22 V (pyrimethamine) and 58 V (quinine), and collision energies were 20 eV (pyrimethamine) and 14 eV (quinine). Capillary voltage was 3 kV, source temperature was 150°C, desolvation temperature was 400°C, and desolvation gas flow was 900 liters per hour. For measurement validation, we performed linearity, correctness, LOD and lower LOQ, repeatability, reproducibility, measurement uncertainty, robustness, and carryover. The detection range was validated between 0.3 and 22 mg/liter. Unbound dolutegravir plasma concentrations were quantified with a validated UPLC-MS/MS bioquantification method as previously described (*61*).

### Inducible HIV-1 reservoir

The frequency of CD4^+^ T cells expressing MS HIV-1 RNA was determined using TILDA with some modifications to the protocol described previously by our group (*68*). Briefly, total CD4^+^ T cells were isolated from PBMCs by negative magnetic selection using EasySep CD4^+^ Human CD4^+^ T Cell Enrichment kit (STEMCELL Technologies). Following isolation, 1.5 × 10^6^ CD4^+^ T cells/ml were rested in complete RPMI 1640 for 5 to 8 hours before 12 hours of stimulation with PMA (100 ng/ml) and ionomycin (1 μg/ml) (both from Sigma-Aldrich). After stimulation, CD4^+^ T cells were washed and resuspended in serum-free RPMI 1640. Cells were counted and serially diluted accordingly: 1.8 × 10^6^ cells/ml, 9 × 10^5^ cells/ml, 3 × 10^5^ cells/ml, and 1 × 10^5^ cells/m. For certain samples with smaller reservoirs, TILDA was performed using a higher input of CD4^+^ T cells (two- to fourfold). In the preamplification reaction, 10 μl of the cell suspension from each dilution was dispensed into 24 to 48 wells of a 96-well plate containing 2 μl of one-step RT-PCR enzyme (Qiagen), 10 μl of 5× one-step RT-PCR buffer (Qiagen), 10 μl of Triton X-100 (0.3%), 0.25 μl of RNAsin (40 U/μl), 2 μl of dNTPs (10 mM each), 1 μl of tat1.4 forward primer (20 μM) and rev reverse primer (20 μM) (as published), and nuclease-free water to a final reaction volume of 50 μl. The one-step RT-PCR was run using the following thermocycling conditions: 50°C for 30 min, 95°C for 15 min, followed by 25 cycles of 95°C for 1 min, 55°C for 1 min, and 72°C for 2 min, and a final extension at 72°C for 5 min. After preamplification, 2 μl of the products was used as input for the real-time PCR to detect tat/rev MS HIV-1 RNA in a final reaction volume of 20 μl, which consisted of 5 μl of 4× TaqMan Fast Advanced Master mix (Thermo Fisher Scientific), 0.4 μl of tat2.0 forward primer, rev reverse primer (each at 20 μM), and MS *tat/rev* probe (5 μM). The real-time PCR was performed using the following program: 50°C for 5 min, 95°C for 20 s, followed by 45 cycles of 95°C for 3 s and 60°C for 30 s. Positive wells at each dilution were recorded and used to determine the frequency of cells expressing tat/rev MS HIV-1 RNA by the maximum likelihood method. The interoperator reproducibility of the assay was evaluated using samples obtained from participants in the pyrimethamine arm (fig. S5, B to D). Primers used for qPCR to generate the end points by TILDA and the other PCR-based assays are listed in table S8.

### Statistical analysis

The sample size was based on the null hypothesis that the change in CA US HIV-1 RNA between the study arms is equal and the alternative hypothesis that the change in CA US HIV-1 RNA is not equal. Assuming a mean change of 31 copies of CA US HIV-1 RNA (SD estimated at 20) by the intervention based on previous trials (*35*), we could detect a twofold increase between any of the four groups with six participants per group at 80% power and alpha 0.05. A twofold change has also been observed in other clinical trials and in our preclinical experiments with pyrimethamine ex vivo (*24*, *34*, *44*). This therefore was considered a realistic target to substantiate the power calculation on. We included seven participants per study arm to account for dropout.

The measurements related to the end points CA US HIV-1 RNA, MS HIV-1 RNA, plasma HIV-1 RNA, and drug plasma levels were described as median with IQR or full range. When the CA US HIV-1 RNA measurement was below the limit of quantitation set at 16 copies per 150 ng of total RNA, we imputed 16 copies at these data points for further calculations. The fold change difference per study arm from pretreatment CA US HIV-1 RNA levels to the time points 6 hours, day 14, and day 42 after first dosing was calculated by adding up the fold change per individual divided by the total number of measurements available per time point. We analyzed the primary end point by a generalized estimating equation model to evaluate whether any difference in the CA US HIV-1 RNA existed at any time point during the trial in any of the three interventional arms compared to the control arm. An interaction term of time and treatment was included. We used the Bliss independence method to conclude on synergism between pyrimethamine and VPA using the following equation μ_(1+2)exp._ = [1 − (1 – μ_1_) × (1 − μ_2_)]. The difference between the observed and expected amount of CA US HIV-1 RNA was calculated, and combination therapy was considered synergistic if the difference and its 95% confidence interval were >0. Because of the number of participants in this pilot, all secondary end points were exploratory. We therefore limited the use of inferential statistics to assessing the median fold changes between the time points in CA US HIV-1 RNA and BAF target genes within and between treatment arms by Wilcoxon signed-rank tests or Mann Whitney *U* tests, and we used Pearson’s test to explore correlations between clinical variables and plasma pyrimethamine levels with the primary end point CA US HIV-1 RNA. We did not adjust for multiple testing post hoc because we powered this pilot study to analyze the primary end point and did not predefine the number of exploratory analyses.
