# Supplementary material for: The BAF complex inhibitor pyrimethamine reverses HIV-1 latency in people with HIV-1 on antiretroviral therapy
Source: Sci Adv. 2023 Mar 15;9(11):eade6675. doi: 10.1126/sciadv.ade6675 (PMC10017042; doi:10.1126/sciadv.ade6675)
Supplement: Supplementary file 1 — Figs. S1 to S4 Tables S1 to S9 [file sciadv.ade6675_sm.pdf]

Science Advances
AAAS

# Supplementary Materials for

## The BAF complex inhibitor pyrimethamine reverses HIV-1 latency in people with HIV-1 on antiretroviral therapy

Henrieke A. B. Prins *et al.*

Corresponding author: Casper Rokx, c.rokx@erasmusmc.nl; Tokameh Mahmoudi, t.mahmoudi@erasmusmc.nl

*Sci. Adv.* **9**, eade6675 (2023)
DOI: 10.1126/sciadv.ade6675

**This PDF file includes:**

Supplementary Text
Figs. S1 to S4
Tables S1 to S9

**SUPPLEMENTARY DATA**

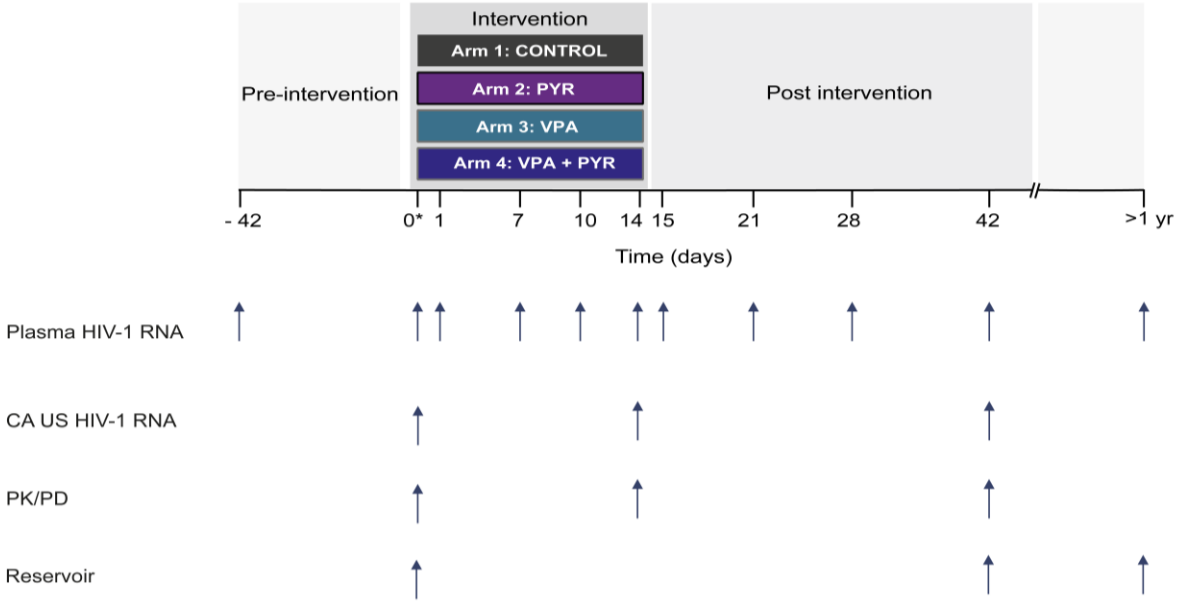


**Fig. S1. Study design overview.** The screening time point is shown at day -42. The three treatment arms and a fourth control arm are shown in blue (VPA arm), purple (PYR arm), dark blue (VPA+PYR), and black (control). Sampling time points for primary and secondary endpoint analysis are indicated by small solid arrows below the timeline. All participants provided 1 extra blood sample at least 1 year after the day 42 visit. * On day 0, CA HIV-1 RNA was measured in samples taken at t=0 hours and after 6 hours. VPA: valproic acid; PYR: pyrimethamine; CA HIV-1 RNA: cell-associated HIV-1 RNA; PK: pharmacokinetics; PD: pharmacodynamics.

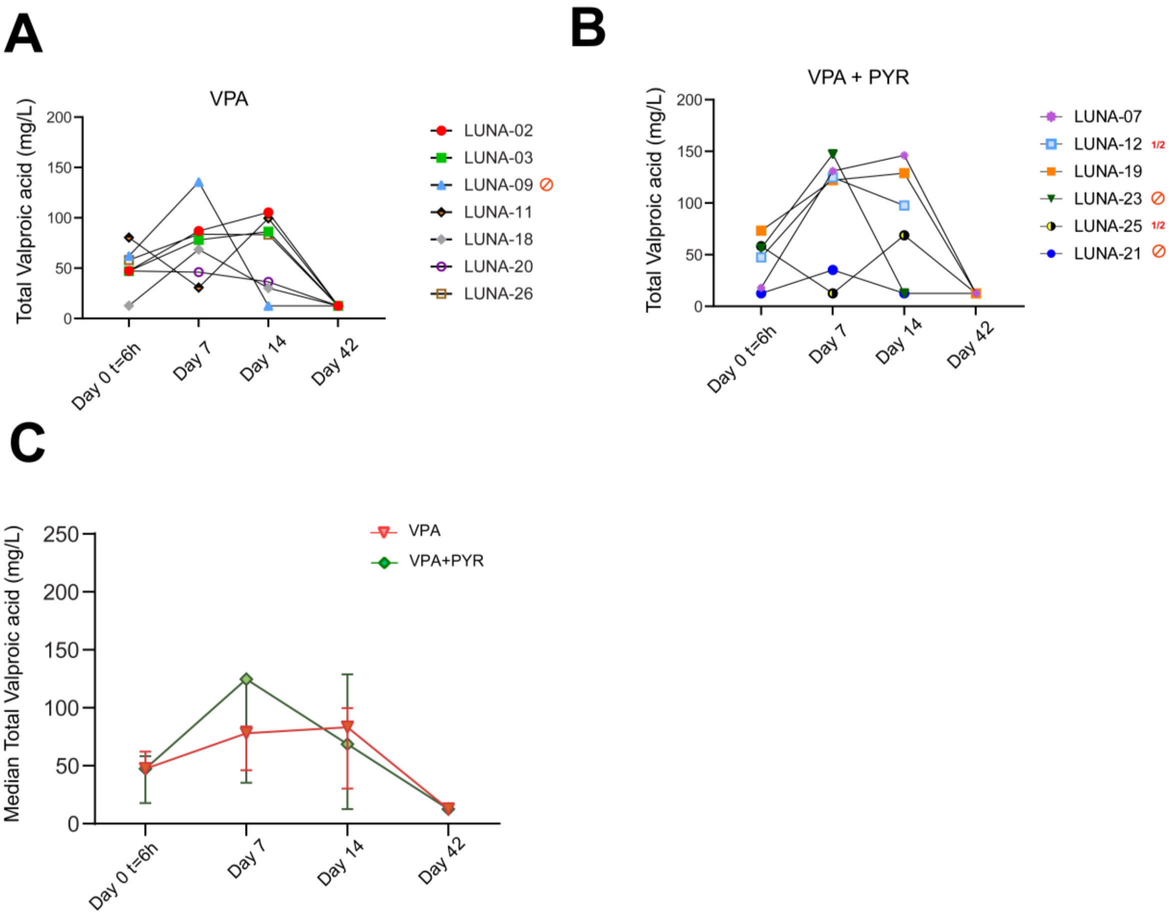


**Fig. S2. Pharmacokinetics of the drug valproic acid in study participants.** (A-B) Pharmacokinetics analysis of the drug valproic acid in study participants represented as plasma valproic acid levels per individual on valproic acid (A) or the combination treatment with pyrimethamine and valproic acid (B) and the median total valproic acid (mg/L) level in plasma (C) including all participants of the valproic acid arm (red) and the combination treatment arm with pyrimethamine and valproic acid (green) at 6 hours after first dosing (day 0, t=6hr ), at day 7, at the end of treatment period (day 14) and 28 days after end of treatment (day 42). Valproic acid concentration measurement was unsuccessful at day 42 for LUNA-12. Participants that stopped study medication or had dose adjustments are indicated with either a red stop sign or red ½ symbol and a red cross on the individual points in the graph. In the valproic acid arm one participant stopped his study medication on day 8 (LUNA-09). In the combination treatment arm, three participants stopped both compounds (LUNA-23 on day 3, LUNA-06 on day 7 and LUNA-21 on day 10), and two participants had their dosage adjusted (LUNA-25 had VPA dose halved from day 2 on, PYR dose halved from day 7 on, and LUNA-12 had PYR dose halved from day 7 on).

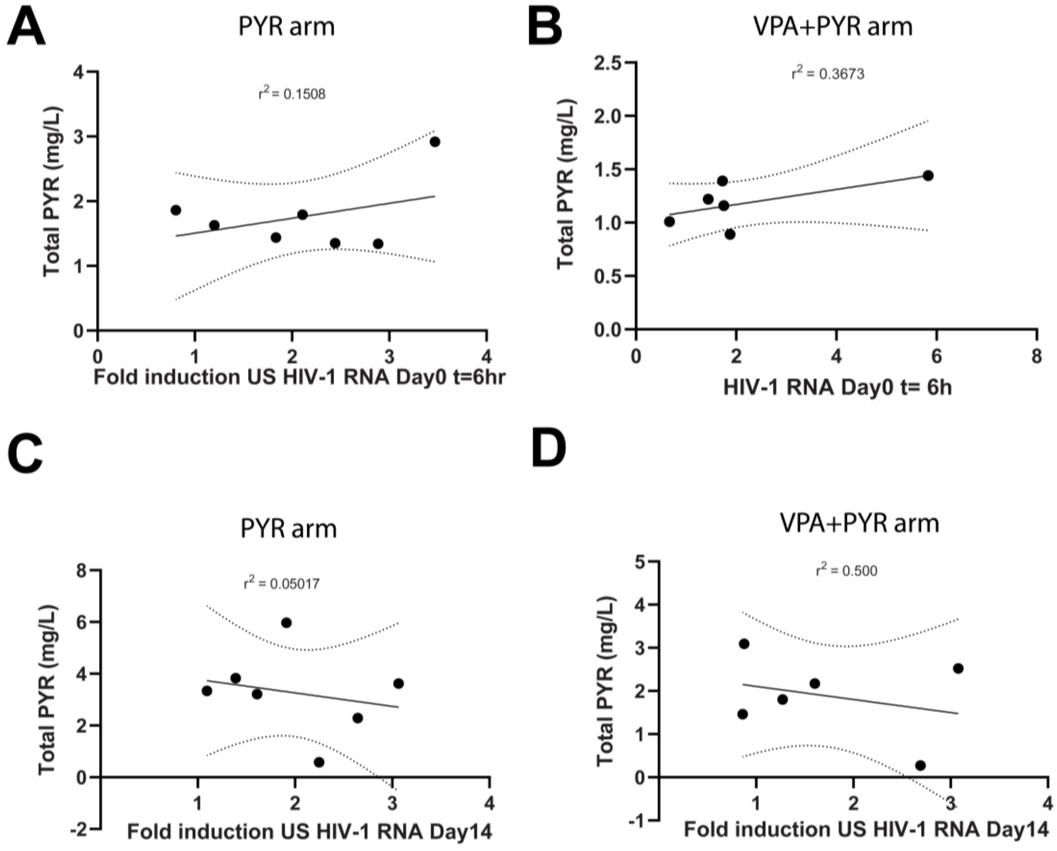


**Fig. S3. Correlation plots of pyrimethamine plasma levels with the primary endpoint cell associated HIV-1 RNA.** Mean and 95% confidence interval correlations with coefficient values (r) between total pyrimethamine (mg/L) and CA US HIV-1 RNA fold change at 6 hours after the first dosing participants on pyrimethamine (A), combination treatment with pyrimethamine and valproic acid (B) and at d14 for these 2 groups (C-D) in 13 of 14 participants exposed to pyrimethamine with sufficient cells available for CA US HIV-1 RNA measurements. Coefficients were calculated by Pearson correlations.

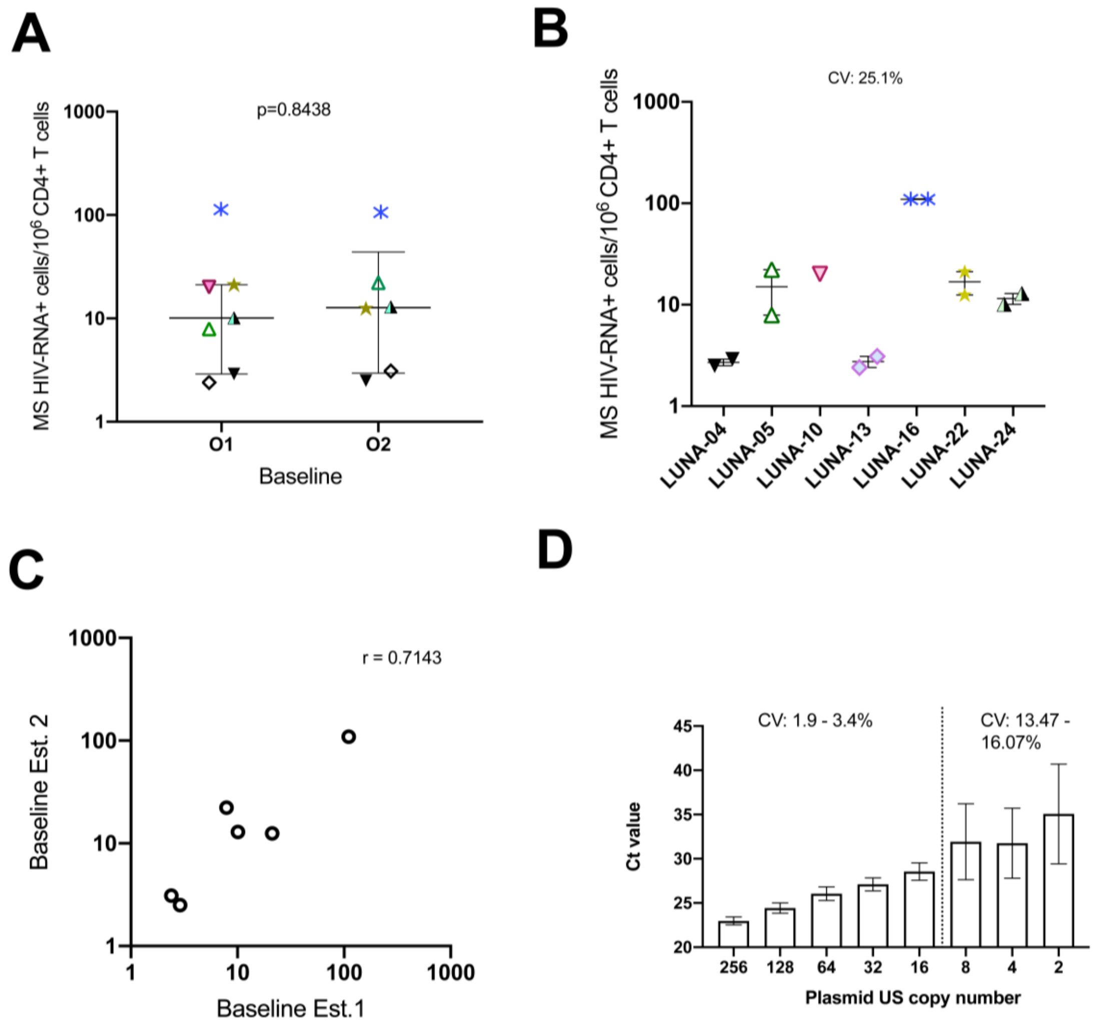


**Fig. S4. Assay reproducibility and standard curve analysis.** (A) and (B) Inter-operator reproducibility of TILDA. Measurements of inducible reservoir size in baseline samples obtained from participants in the pyrimethamine arm. TILDA was performed by different operators [O1 and O2] in independent experiments. The error bars represent the interquartile range. A Wilcoxon matched-pairs signed rank test was used to compare the medians of the TILDA data generated by O1 and O2. CV indicates coefficient of variation. (C) Spearman correlation of the TILDA data between operators. (D) Standard curve interpolation of absolute number of US copies ranging from 2 to 256 copies of a plasmid containing the full-length HIV-1 genome (pNL4.3.Luc.R-E-) and cycle threshold value (Ct value) in the PCR. CV indicates coefficient of variation. Based on standard curve analysis we assigned a limit of quantification (LOQ) of 16 copies with an intra assay coefficient of variation of <5%.

**Table S1. Total number of self-reported adverse events and their severity during trial participation**

| | Grade 1 | Grade 2 | Grade3 | Any Grade |
|---|---|---|---|---|
| Control arm | 4 | 0 | 0 | 4 |
| Pyrimethamine arm | 33 | 3 | 2 | 38 |
| Valproic acid arm | 13 | 2 | 0 | 15 |
| Valproic acid + pyrimethamine arm | 32 | 13 | 1 | 46 |
| **Total adverse events** | 82 | 18 | 3 | 103 |
| Presumed related | 72 | 16 | 3 | 91 |
| Presumed not related | 10 | 2 | 0 | 12 |

**Table S2. Total number of self-reported adverse events (AE) per treatment arm during trial participation**

| AE considered related | PYR | VPA | PYR+VPA | Control |
|---|---|---|---|---|
| **Gastrointestinal** | 9 | 5 | 17 | 0 |
| Nausea | 4 | 2 | 6 | |
| Vomiting | 3 | 3 | 6 | |
| Diarrhea | 1 | | 1 | |
| Dysgeusia | 1 | | | |
| Dyspepsia | | | 2 | |
| Early satiety | | | 1 | |
| Bloating | | | 1 | |
| **Neurological** | 12 | 7 | 21 | 1 |
| Vertigo | | 1 | 5 | |
| Headache | 2 | 2 | 2 | |
| Somnolence | | 1 | 4 | |
| Involuntary muscle movement | | 1 | | |
| Lethargy | | 1 | 2 | |
| Dizziness | 1 | 1 | | |
| Insomnia | 3 | | 1 | |
| Presyncope | 1 | | | 1 |
| Syncope | 2 | | 1 | |
| Light-headedness | 1 | | 2 | |
| Tremor | 1 | | | |
| Dysarthria | 1 | | | |
| Tinnitus | | | 2 | |
| Agitation | | | 1 | |
| Hypersomnia | | | 1 | |
| **Other organ systems** | 12 | 1 | 6 | 0 |
| Sweats | 1 | | | |
| Rash | 2 | | | |
| Nosebleed | 1 | | | |
| Pruritus | 1 | | 1 | |
| Cold extremities | 1 | | 1 | |
| Malaise | 2 | | 1 | |
| Urine frequency | | | 1 | |
| Edema limbs | | 1 | | |
| Backache | | | 1 | |
| Anemia | 1 | | | |
| Neutropenia | 1 | | 1 | |
| Thrombocytopenia | 2 | | | |
| **Any related** | **33** | **13** | **44** | **1** |
| **AE considered not related** | | | | |
| Sore throat | 1 | | | |
| Nausea | 1 | | | |
| Vomiting | 1 | | | 1 |
| Diarrhea | | | | 1 |
| Flu-like symptoms | | | | 1 |
| Backache | 1 | | | |
| Ache right upper arm | 1 | | | |
| Headache | | 1 | | |
| Disturbed sleep | | 1 | | |
| Hyperglycemia | | | 1 | |
| Pharyngitis | | | 1 | |
| **Any non-related** | **5** | **2** | **2** | **3** |

**Table S3. Total number of self-reported adverse events (AE) and their severity during trial participation**

| AE considered related | Grade 1 N(%) | Grade 2 N(%) | Grade3 N(%) | Any Grade N(%) | No. of patients N(%) |
|---|---|---|---|---|---|
| Malaise | 2 | 1 | 0 | 3 | 3 |
| Nausea | 8 | 4 | 0 | 12 | 12 |
| Dyspepsia | 2 | 0 | 0 | 2 | 2 |
| Early satiety | 1 | 0 | 0 | 1 | 1 |
| Bloating | 1 | 0 | 0 | 1 | 1 |
| Vomiting | 11 | 1 | 0 | 12 | 9 |
| Diarrhea | 2 | 0 | 0 | 2 | 2 |
| Headache | 5 | 1 | 0 | 6 | 6 |
| Somnolence | 5 | 0 | 0 | 5 | 4 |
| Hypersomnia | 1 | 0 | 0 | 1 | 1 |
| Insomnia | 4 | 0 | 0 | 4 | 4 |
| Involuntary muscle movement | 1 | 0 | 0 | 1 | 1 |
| Vertigo | 3 | 3 | 0 | 6 | 5 |
| Tinnitus | 1 | 1 | 0 | 2 | 2 |
| Lethargy | 2 | 1 | 0 | 3 | 3 |
| Agitation | 0 | 1 | 0 | 1 | 1 |
| Dizziness | 2 | 0 | 0 | 2 | 2 |
| Light headedness | 3 | 0 | 0 | 3 | 3 |
| Presyncope | 2 | 0 | 0 | 2 | 2 |
| Syncope | 0 | 0 | 3 | 3 | 3 |
| Edema limbs | 1 | 0 | 0 | 1 | 1 |
| Cold extremities | 2 | 0 | 0 | 2 | 2 |
| Sweats | 1 | 0 | 0 | 1 | 1 |
| Rash | 2 | 0 | 0 | 2 | 1 |
| Pruritus | 2 | 0 | 0 | 2 | 2 |
| Dysarthria | 1 | 0 | 0 | 1 | 1 |
| Dysgeusia | 1 | 0 | 0 | 1 | 1 |
| Tremor | 1 | 0 | 0 | 1 | 1 |
| Urinary frequency | 1 | 0 | 0 | 1 | 1 |
| Back pain | 1 | 0 | 0 | 1 | 1 |
| Nosebleed | 1 | 0 | 0 | 1 | 1 |
| Anemia | 1 | 0 | 0 | 1 | 1 |
| Platelet count decreased | 1 | 1 | 0 | 2 | 1 |
| Neutrophil count decreased | 0 | 2 | 0 | 2 | 2 |
| **Any related AE** | 72 | 16 | 3 | 91 | 84 |
| **AE considered not related** | | | | | |
| Nausea | 1 | 0 | 0 | 1 | 1 |
| Vomiting | 2 | 0 | 0 | 2 | 2 |
| Diarrhea | 1 | 0 | 0 | 1 | 1 |
| Headache | 1 | 0 | 0 | 1 | 1 |
| Disturbed sleep | 1 | 0 | 0 | 1 | 1 |
| Ache right upper arm | 0 | 1 | 0 | 1 | 1 |
| Upper back ache | 1 | 0 | 0 | 1 | 1 |
| Sore throat | 1 | 0 | 0 | 1 | 1 |
| Hyperglycemia | 0 | 1 | 0 | 1 | 1 |
| Pharyngitis | 1 | 0 | 0 | 1 | 1 |
| Flu like symptoms | 1 | 0 | 0 | 1 | 1 |
| Any not related | 10 | 2 | 0 | 12 | 12 |
| **Total AE** | **82** | **18** | **3** | **103** | **96** |

**Table S4. Baseline characteristics of participants with and without adjusted interventions**

| Baseline Characteristics | With treatment adjustment n =28 | Without treatment adjustment N=7 |
|---|---|---|
| Male | 7 (100) | 21 (100) |
| Ethnic origin | | |
| -White European | 5 (71.4) | 19 (90.5) |
| -Latin American or Hispanic | 1 (14.3) | 2 (9.5) |
| -Black Caribbean | 1 (14.3) | 0 |
| Age (years) | 51 (42-58) | 54 (50-61) |
| HIV subtype B[a] | 6 (85.7) | 18 (85.7) |
| History of AIDS | 2 (28.6) | 6 (28.6) |
| Years from HIV diagnosis until inclusion | 7.6 (6.2-8.0) | 10.7 (7.6-18.5) |
| Years on cART | 7.2 (4.7-7.3) | 8.5 (5.6-12.3) |
| Years with HIV-RNA <50 copies per mL | 5.1 (4.5-7.1) | 7.2 (5.1-11.8) |
| Initiated cART during acute HIV infection | 0 | 3 (14.3) |
| Pre-cART plasma viral load zenith $\log_{10}$ copies per mL | 4.81 (4.58-5.04) | 4.90 (4.77-5.35) |
| Pre-cART nadir CD4+ T-cell count per µL | 300 (205-420) | 220 (140-290) |
| CD4+ T-cell count per µL at inclusion | 750 (580-930) | 650 (530-790) |
| cART | | |
| -NNRTI based[b] | 3 (42.9) | 13 (61.9) |
| -INSTI based[c] | 4 (57.1) | 8 (38.1) |

*Data are number with percentage or median with interquartile ranges.*
*[a]Other HIV-1 subtypes include CRF01-AE (n=2), CRF01-AG (n=1), C (n=1).*
*[b]NNRTI were rilpivirine (n=6), nevirapine (n=6), efavirenz (n=4). 15 NRTI backbones consisted of emtricitabine (FTC) and either tenofovir disoproxil fumarate (TDF) or tenofovir alafenamide (TAF) and 1 NRTI backbone consisted of abacavir with lamivudine.*
*[c]INSTI were dolutegravir (n=10) and elvitegravir/cobicistat (n=2). 7 NRTI backbones consisted of FTC and either TDF or TAF, 3 NRTI backbones consisted of lamivudine (3TC) and abacavir (ABC); and 2 had dolutegravir with 3TC as NRTI backbone.*
*Abbreviations: cART: combination antiretroviral therapy; INSTI: integrase strand transfer inhibitor; NNRTI: non-nucleoside reverse transcriptase inhibitor.*

**Table S5. Individual clinical characteristics and cell associated unspliced HIV-1 RNA during LUNA.**

| | CD4 T-cell count per µL at inclusion | Years on cART | cART | CA US HIVRNA copies/150 ng Total RNA Day 0, pretreatment | CA US HIVRNA copies/150ng Total RNA Day 0, 6 hours | CA US HIVRNA copies/150 ng Total RNA Day 14 | CA US HIVRNA copies/150 ng Total RNA Day 42 |
|---|---|---|---|---|---|---|---|
| **PYR** | | | | | | | |
| LUNA-04 | 580 | 7,3 | FTC, TAF, EVG/c | 32,60 | 94,04 | 62,36 | 101,16 |
| LUNA-05 | 390 | 5,5 | FTC, TDF, EFV | 79,98 | 96,01 | 111,26 | 84,39 |
| LUNA-10 | 810 | 7,2 | FTC, TAF, NVP | 51,15 | 125,01 | 114,91 | 85,80 |
| LUNA-13 | 850 | 5,6 | FTC, TDF, EFV | 68,92 | 126,50 | 111,02 | 54,17 |
| LUNA-16 | 400 | 16,3 | FTC, TDF, NVP | 24,35 | 51,31 | 74,67 | 82,71 |
| LUNA-22 | 610 | 7,7 | FTC, TAF, DTG | 49,43 | 39,82 | 54,10 | 69,81 |
| LUNA-24 | 730 | 23,1 | FTC, TAF, NVP | 16,00 | 55,52 | 42,33 | 29,28 |
| **PYR+VPA** | | | | | | | |
| LUNA-06[1] | 630 | 7,2 | FTC, TAF, RPV | | | | |
| LUNA-07 | 960 | 12,2 | FTC, TDF, RPV | 53,68 | 35,85 | 46,27 | 28,10 |
| LUNA-12 | 1050 | 3,5 | 3TC, ABC, DTG | 34,27 | 49,48 | 54,91 | |
| LUNA-19 | 240 | 5,6 | FTC, TAF, RPV | 32,97 | 57,84 | 28,92 | |
| LUNA-21 | 420 | 4,8 | FTC, TDF, RPV | 28,25 | 48,73 | 35,93 | 45,78 |
| LUNA-23 | 530 | 7,5 | 3TC, ABC, DTG | 16,64 | 97,05 | 51,25 | 49,58 |
| LUNA-25 | 1180 | 4,5 | 3TC, DTG | 21,83 | 41,01 | 58,74 | 31,47 |
| **VPA** | | | | | | | |
| LUNA-02 | 790 | 4,8 | FTC, TAF, RPV | 95,16 | 101,51 | 161,79 | 59,33 |
| LUNA-03 | 590 | 7,6 | FTC, TAF, DTG | 12,93 | 10,16 | 18,41 | 16,00 |
| LUNA-09 | 750 | 7,4 | FTC, TAF, DTG | 22,90 | 42,74 | 28,40 | 49,74 |
| LUNA-11 | 1250 | 21,1 | FTC, TDF, NVP | 39,10 | 53,04 | 97,80 | 59,03 |
| LUNA-18 | 530 | 12,3 | FTC, TDF, NVP | 16,00 | 19,23 | 16,00 | 16,00 |
| LUNA-20 | 830 | 4,3 | FTC, TDF, EFV | 76,70 | 51,59 | 47,89 | 52,98 |

| | | | | | | | |
|---|---|---|---|---|---|---|---|
| LUNA-26 | 330 | 9,3 | FTC, TAF, DTG | 16,00 | 16,00 | 18,82 | 22,83 |
| **CONTROL** | | | | | | | |
| LUNA-01 | 780 | 3,9 | FTC, TDF, RPV | 16,00 | 16,00 | 16,00 | 16,00 |
| LUNA-08 | 700 | 14,7 | FTC, TDF, EFV | 50,82 | 63,21 | 57,75 | 45,16 |
| LUNA-14 | 650 | 7,4 | 3TC, DTG | 120,00 | 114,52 | 93,02 | 100,41 |
| LUNA-15 | 680 | 21,7 | FTC, TAF, EVG/c | 62,96 | 113,72 | 113,54 | 120,88 |
| LUNA-17 | 530 | 8,5 | 3TC, ABC, NVP | 16,00 | 39,37 | 29,24 | 22,56 |
| LUNA-27 | 980 | 9,8 | FTC, TDF, DTG | 16,00 | 16,00 | 16,00 | 16,00 |
| LUNA-28 | 470 | 11,2 | 3TC, ABC, DTG | 16,00 | 16,00 | 16,00 | 16,00 |

[1] LUNA-06 had insufficient PBMC at day 0 at 6 hours post dosing to measure the primary endpoint. Due to the missing baseline measurement, no CA US HIV-1 RNA were measured at the other timepoints. *Abbreviations: cART: combination antiretroviral therapy; PYR: pyrimethamine; VPA: valproic acid; FTC: emtricitabine; 3TC: lamivudine; ABC: abacavir; TDF: tenofovir disoproxil fumarate; TAF: tenofovir alafenamide; EFV: efavirenz; NVP: nevirapine; RPV: rilpivirine; DTG: dolutegravir; EVG/c: elvitegravir/cobicistat;* .

**Table S6: plasma HIV-1 RNA evolution during LUNA per participant.**

| | **Day 0, 0hr** | **Day 0, 6hr** | **Day 1** | **Day 7** | **Day 10** | **Day 14** | **Day 15** | **Day 21** | **Day 28** | **Day 42** |
|---|---|---|---|---|---|---|---|---|---|---|
| **PYR** | | | | | | | | | | |
| LUNA-04 | **<20/P** | * | **<20/N** | **<20/N** | **<20/N** | **<20/P** | **<20/N** | **<20/P** | **<20/N** | **<20/P** |
| LUNA-05 | **<20/N** | * | **<20/P** | **<20/N** | **<20/N** | **<20/N** | **<20/N** | **<20/N** | **<20/P** | **<20/N** |
| LUNA-10 | **<20/N** | * | **<20/N** | **<20/N** | **<20/N** | **<20/P** | **<20/N** | **<20/P** | **<20/P** | **<20/N** |
| LUNA-13 | **<20/P** | **<20/N** | **<20/N** | **<20/N** | **<20/P** | **<20/N** | **<20/N** | **<20/N** | **<20/N** | **<20/P** |
| LUNA-16 | **<20/N** | **<20/N** | **<20/N** | **<20/P** | **<20/N** | **<20/N** | **<20/N** | **<20/N** | **20** | **<20/N** |
| LUNA-22 | **<20/N** | **<20/P** | **<20/N** | **<20/N** | **<20/N** | **<20/N** | **<20/P** | **<20/N** | **<20/N** | **<20/N** |
| LUNA-24 | **<20/P** | **<20/P** | **<20/P** | **<20/N** | **<20/N** | **<20/N** | **<20/N** | **<20/N** | **<20/N** | **<20/N** |
| **PYR+VPA** | | | | | | | | | | |
| LUNA-06 | **<20/N** | * | **<20/N** | **<20/N** | **<20/N** | **<20/N** | **<20/N** | **<20/N** | **<20/N** | **<20/N** |
| LUNA-07 | **<20/N** | * | **<20/N** | **<20/P** | **<20/N** | **<20/N** | **<20/N** | **<20/P** | **<20/P** | **<20/N** |
| LUNA-12 | **<20/N** | * | **<20/P** | **<20/N** | **<20/N** | **<20/N** | **<20/N** | **<20/N** | **<20/N** | **<20/N** |
| LUNA-19 | **<20/N** | **<20/N** | **<20/N** | **<20/N** | **<20/N** | **<20/N** | **<20/N** | **<20/N** | **<20/N** | **<20/P** |
| LUNA-21 | **<20/N** | **<20/N** | **<20/N** | **<20/N** | **<20/N** | **<20/N** | **<20/N** | **<20/N** | **<20/N** | **<20/N** |
| LUNA-23 | **<20/P** | **<20/N** | **<20/P** | **22** | **<20/P** | **<20/N** | **<20/P** | **<20/P** | **<20/N** | **<20/P** |
| LUNA-25 | **<20/N** | **<20/N** | **<20/P** | **<20/N** | **<20/P** | **<20/N** | **<20/P** | **<20/N** | **<20/N** | **<20/P** |
| **VPA** | | | | | | | | | | |
| LUNA-02 | **<20/N** | * | **<20/N** | **<20/N** | **<20/N** | **<20/N** | **<20/P** | **<20/N** | **<20/N** | **<20/N** |
| LUNA-03 | **<20/N** | * | **<20/N** | **<20/N** | **<20/N** | **<20/N** | **<20/N** | **<20/N** | **<20/N** | **<20/N** |
| LUNA-09 | **<20/N** | * | **<20/P** | **<20/N** | **<20/N** | **<20/N** | **<20/N** | **<20/N** | **<20/N** | **<20/P** |
| LUNA-11 | **<20/N** | * | **<20/N** | **<20/N** | **<20/N** | **<20/P** | **<20/N** | **<20/P** | **<20/N** | **<20/N** |
| LUNA-18 | **<20/N** | **<20/N** | **<20/N** | **<20/N** | **<20/N** | **<20/N** | **<20/N** | **<20/N** | **<20/N** | **<20/P** |
| LUNA-20 | **<20/P** | **<20/N** | **41** | **<20/P** | **<20/N** | **<20/P** | **<20/P** | **<20/P** | **<20/N** | **<20/P** |
| LUNA-26 | **<20/N** | **<20/P** | **<20/N** | **<20/N** | **<20/N** | **<20/N** | **<20/P** | **21** | **<20/N** | **<20/P** |
| **CONTROL** | | | | | | | | | | |
| LUNA-01 | **<20/N** | * | **<20/N** | **<20/N** | **<20/P** | **<20/N** | **<20/N** | **<20/N** | **<20/N** | **<20/N** |
| LUNA-08 | **<20/N** | * | **<20/N** | **<20/N** | **<20/P** | **<20/P** | **<20/N** | **<20/P** | **<20/P** | **<20/N** |
| LUNA-14 | **<20/N** | **<20/N** | **<20/N** | **<20/P** | **<20/N** | **<20/P** | **<20/N** | **<20/P** | **<20/P** | **<20/N** |
| LUNA-15 | **<20/N** | **<20/N** | **<20/P** | **<20/N** | **<20/N** | **<20/N** | **<20/N** | **<20/N** | **<20/N** | **<20/N** |
| LUNA-17 | **<20/N** | **<20/N** | **<20/N** | **<20/N** | **<20/N** | **<20/P** | **<20/N** | **<20/N** | **<20/N** | **<20/P** |
| LUNA-27 | **<20/N** | **<20/N** | **<20/N** | **<20/N** | **<20/N** | **<20/N** | **<20/N** | **<20/N** | **<20/N** | **<20/P** |
| LUNA-28 | **<20/N** | **<20/N** | **<20/P** | **<20/N** | **<20/P** | **<20/P** | **<20/N** | **<20/P** | **<20/N** | **<20/N** |

*Protocol violation where day 0 6 hour timepoint was not collected for participants LUNA-01 till LUNA-12.
/N denotes target viral genome not detected below level of quantitation.
/P denotes target viral genome detected below level of quantitation.
Those with and without pyrimethamine exposure were comparable with regard to the proportions of samples with plasma HIV-1 RNA above the assay detection limit both during routine care prior to study inclusion since cART initiation (20.7% versus 17.8%) and during the study at the fixed measurements (22.2% versus 23.6%).
*Abbreviations: PYR: pyrimethamine; VPA: valproic acid.*

**Table S7: Fold-induction gene expression values for BAF target genes**

| | *Fold-induction in IL-10 gene expression at Day 0, 6 hours relative to Day 0, pretreatment* | *Fold-induction in SOCS3 gene expression at Day 0, 6 hours relative to Day 0, pretreatment* | *Fold-induction in CBX7 gene expression at Day 0, 6 hours relative to Day 0, pretreatment* | *Fold-induction in B-ACTIN gene expression at Day 0, 6 hours relative to Day 0, pretreatment* |
|---|---|---|---|---|
| **PYR** | | | | |
| LUNA-04 | 2.24 | 4.59 | 1.25 | 0.86 |
| LUNA-05 | 6.4 | 3.27 | 1.03 | 1.04 |
| LUNA-10 | 2.54 | 2.52 | 1.68 | 1.29 |
| LUNA-13 | 2.74 | 2.27 | 1.12 | 1.51 |
| LUNA-16 | 1.48 | 0.60 | 12.42 | 0.82 |
| LUNA-22 | 1.74 | 5.35 | 4.23 | 0.94 |
| LUNA-24 | 6.66 | 1.14 | 2.02 | 0.94 |
| **PYR+VPA** | | | | |
| LUNA-07 | 0.53 | 1.96 | 0.87 | 0.87 |
| LUNA-12 | 0.86 | 0.94 | 0.70 | 0.85 |
| LUNA-19 | 1.99 | 1.03 | 1.11 | 1.14 |
| LUNA-21 | 3.31 | 0.93 | 0.79 | 0.65 |
| LUNA-23 | 3.81 | 2.48 | 1.25 | 0.8 |
| LUNA-25 | 0.61 | 0.78 | 1.73 | 1.58 |
| **VPA** | | | | |
| LUNA-02 | 0.42 | 0.53 | 0.82 | 0.68 |
| LUNA-03 | 0.64 | 0.67 | 1.71 | 1.12 |
| LUNA-09 | NA | 0.52 | 1.16 | 2.5 |
| LUNA-11 | 1.25 | 0.38 | 0.9 | 0.94 |
| LUNA-18 | 1.10 | 1.92 | 0.54 | 0.9 |
| LUNA-20 | 0.94 | 0.85 | 0.23 | 0.69 |
| LUNA-26 | 1.87 | 2.47 | 0.87 | 0.61 |
| **CONTROL** | | | | |
| LUNA-01 | NA | NA | NA | 0.92 |
| LUNA-08 | 0.72 | 0.02 | 0.83 | NA |
| LUNA-14 | 1.16 | 0.52 | 0.69 | NA |
| LUNA-15 | 0.20 | 1.00 | 1.53 | NA |
| LUNA-17 | 2.49 | 0.59 | 1.89 | NA |
| LUNA-27 | NA | 0.54 | 0.44 | 0.94 |
| LUNA-28 | 0.36 | 0.88 | 1.18 | 1.34 |

NA denotes not available due to insufficient sample. *Abbreviations: PYR: pyrimethamine; VPA: valproic acid.*

**Table S8: TILDA reservoir as number of cells with multiply spliced HIV-RNA detectable per million CD4+ T-cells per participant.**

| | **Day 0, 0 hour** | **Day 42** | **>1year** |
|---|---|---|---|
| **PYR** | | | |
| LUNA-04 | **2.7** | **1.6** | **NA** |
| LUNA-05 | **15.1** | **10** | **22.2** |
| LUNA-10 | **20.1** | **22.9** | **15.2** |
| LUNA-13 | **2.8** | **8.8** | **19.3** |
| LUNA-16 | **109.8** | **122.8** | **91.8** |
| LUNA-22 | **16.9** | **22.8** | **13.9** |
| LUNA-24 | **11.5** | **9.0** | **6.1** |
| **PYR+VPA** | | | |
| LUNA-06 | **NA (low PBMC)** | | |
| LUNA-07 | **NA (low PBMC)** | | |
| LUNA-12 | **<LOD** | **<LOD** | **0.6** |
| LUNA-19 | **26.5** | **63.0** | **NA** |
| LUNA-21 | **19.0** | **3.0** | **2.0** |
| LUNA-23 | **NA (subtype AG)** | **NA** | **NA** |
| LUNA-25 | **7.3** | **18.3** | **45.7** |
| **VPA** | | | |
| LUNA-02 | **12.5** | **12.7** | **NA** |
| LUNA-03 | **2.5** | **1.2** | **2.2** |
| LUNA-09 | **17.1** | **12.9** | **19.0** |
| LUNA-11 | **3.4** | **NA** | **2.5** |
| LUNA-18 | **NA (subtype C)** | | |
| LUNA-20 | **100.7** | **26.0** | **25.5** |
| LUNA-26 | **326.5** | **NA** | **286.2** |
| **CONTROL** | | | |
| LUNA-01 | **NA (subtype AE)** | | |
| LUNA-08 | **10.2** | **16.2** | **11.5** |
| LUNA-14 | **53.0** | **32.3** | **53.5** |
| LUNA-15 | **131.4** | **394.6** | **338.1** |
| LUNA-17 | **NA (low PBMC)** | | |
| LUNA-27 | **NA (subtype AE)** | | |
| LUNA-28 | **52.7** | **57.8** | **NA** |

No TILDA were performed in participants with missing day 0 measurements due to primer mismatches or low CD4+T-cell yields.
NA denotes not available due to primer mismatches or low CD4+T-cell yields.
*Abbreviations: PYR: pyrimethamine; VPA: valproic acid.*

**Table S9: List of primers used for PCR for CA US HIV-1 RNA, TILDA and BAF complex target genes**

| Primer name | Sequence 5’-3’ |
|---|---|
| US Forward | TCAGCCCAGAAGTAATACCCATGT |
| US Reverse 1 | TGCTATGTCAGTTCCCCTTGGTTCTCT |
| US Reverse 2 | CACTGTGTTTAGCATGGTGTTT |
| US Probe | [6FAM]ATTATCAGAAGGAGCCACCCCACAAGA[BHQ1] |
| IL-10 Forward | GAGTCCTTGCTGGAGGACTTT |
| IL-10 Reverse | CACGGCCTTGCTCTTGTTTT |
| CBX7 Forward | GAGAAGGAGGAGAGAGACCGA |
| CBX7 Reverse | CCCTTGTCCACCAGCTCAG |
| SOCS3 Forward | CCAAGGACGGAGACTTCGAT |
| SOCS3 Reverse | GGTACTCGCTCTTGGAGCTG |
| B-ACTIN Forward | CACAGGGGAGGTGATAGCAT |
| B-ACTIN Reverse | TCAAGTTGGGGGACAAAAAG |
| Cyclophilin A Forward | TCATCTGCACTGCCAAGACTG |
| Cyclophilin A Reverse | CATGCCTTCTTTCACTTTGCC |
| Tat 1.4 | TGG CAG GAA GAA GCG GAG A |
| Tat 2.0 | ACAGTCAGACTCATCAAGTTTCTCTATCAAAGCA |
| Rev | GGATCTGTCTCTGTCTCTCTCTCCACC |
| TILDA Probe | 5’-/56-FAM/TTCCTTCGG/ZEN/GCCTGTCGGGTCCC/3IABkFQ/-3 |

**Study protocol synopsis**
**Protocol version 3.0**
**27 January, 2020**

**Rationale**
The retrovirus HIV integrates as proviral DNA in the genome of our CD4+ T cells. A subset forms a reservoir of latently infected long-lived memory T-cells with nearly absent HIV-DNA transcription. This persistent latent HIV reservoir is the major obstacle for a cure. HIV latency is sustained by multiple host factors that restrict the viral promotor and expression of the viral genome. Latency reversing agents (LRA) can remove these restrictive components and mediate HIV latency reversal. LRA monotherapy with histone deacetylase inhibitors (HDACi), including valproic acid, vorinostat, romidepsin, panobinostat, reactivates HIV but seems insufficient to eliminate the reservoir in vivo.

Our research group has identified the BAF complex as a repressive factor that maintains HIV latency. We investigated the activity of a panel of recently identified small molecule inhibitors of BAF (BAFi) as a new LRA group and showed that BAFi, including the clinically approved drug pyrimethamine at tolerable concentrations, are capable of reversing HIV latency and act synergistic with HDACi in vitro and in CD4+ T cells obtained from people with HIV on suppressive antiretroviral therapy. This offers new opportunities for cure research. We want to conduct the first study with BAFi and assess the potential synergism of 2 LRA with different modes of action on the reservoir in people with HIV.

**Design and primary objective**
The LUNA study is a 6 week prospective, open label, randomized controlled clinical trial. The primary objective is the longitudinal assessment of the BAFi pyrimethamine and of the HDACi valproic acid on the HIV reservoir in people with HIV on antiretroviral therapy.

The main hypothesis tested is:
H0: the change in CA-US HIVRNA between the groups during treatment are equal.
H1: the change in CA-US HIVRNA between the groups during treatment are not equal.

**Study population**
<u>Inclusion criteria</u>
1. HIV-1 infected patients ≥18 years.
2. WHO performance status 0 or 1.
3. Confirmed HIV-1 infection by 4th generation ELISA, Western Blot or PCR.
4. Wild type HIV infection or polymorphisms associated with at highest low-level resistance to any class of ART according to Stanford HIV drug resistance database. Transmitted mutations and acquired mutations due to virological failure associated with resistance of at highest low-level resistance are allowed.
5. On cART.
6. Current plasma HIV-RNA <50 copies/mL for at least 365 days and measured on at least 2 occasions of which at least 1 must be obtained within 365 and 90 days prior to study entry.
7. Current CD4 count at study entry of ≥200 cells/mm3.
8. Pre-cART HIV-RNA ≥10.000 copies/mL.

Exclusion criteria

A potential subject who meets any of the following criteria will be excluded from participation in this study.

1. Previous virological failure, defined as either acquired resistance mutations (>low level resistance) on cART or HIV-RNA >1000 copies/mL on two consecutive measurements during cART.
2. Uncontrolled hepatitis B or C co-infection.
3. Prior exposure to any HDACi, BAFi or other known LRA.
4. Prior exposure to cytotoxic myeloablative chemotherapy for hematological malignancies during cART.
5. Concurrent exposure to strong interacting medication on glucuronidation.
6. Exposure within 90 days prior to study entry to immunomodulators, cytokines, systemic antifungals, dexamethasone, vitamin K antagonists, anti-epileptics, antipsychotica, carbapenems, mefloquine, colestyramine, Any documented opportunistic infection related to HIV in the last 90 days.
7. Inadequate blood counts, renal and hepatic function tests.

a. Haemoglobin <6.5 mmol/L (males) or <6.0 mmol/L (females), leucocytes <2.5 x109/L, absolute neutrophil count <1000 cells/mm3, thrombocytes <100 x109/L, international standardized ratio >1.6, activated partial thromboplastin time >40 seconds.

b. Estimated glomerular filtration rate <50 mL/min (CKD-EPI).

c. ALAT or total bilirubin >2.5x upper limit of normal.

d. All laboratory values must be obtained within 42 days prior to the baseline visit.

8. Megaloblastic anemia due to folate deficiency.
9. Pancreatitis in last 6 months, or chronic pancreatitis.
10. Active malignancy during the past year with the exception of basal carcinoma of the skin, stage 0 cervical carcinoma, Kaposi Sarcoma treated with cART alone, or other indolent malignancies.
11. Females in the reproductive age cannot participate. Males cannot participate if they refuse to abstain from sex or condom use in serodiscordant sexual contact during the study, except if their sexual partner(s) use PREP.
12. Patients with active substance abuse or registered allergies to the investigational medical products.
13. Last, any other condition (familial, psychological, sociological, geographical) which in the investigator’s opinion poses an unacceptable risk or would hamper compliance with the study protocol and follow up schedule, will prohibit participation.

For hepatitis B: patients should be vaccinated, or on pre-exposure prophylaxis through the use of lamivudine/emtricitabine or tenofovir in their cART. Otherwise, standard serological testing should be available within the last 365 days for men with HIV who have sex with men. For other persons with HIV, there should be at least one negative hepatitis B test (either by serology or PCR) For men with HIV who have sex with men, a negative hepatitis C IgG, HCV antigen, blot or HCV-RNA PCR should be available within the previous 365 days. For other persons with HIV, there should be at least one negative hepatitis C test (either IgG, blot or PCR) available.

**Primary endpoint**

The change in HIV reactivation in the reservoir in vivo at treatment initiation and at the end of treatment, measured as the change in cell associated HIV-RNA. The change in reactivation is compared between the

treatment arms. The primary outcome measure is the change in cell associated HIV-RNA between treatment initiation (week 0) and at the end of study (week 6).

**Safety definitions**

The study was terminated in case of excessive serious adverse events based on predefined safety criteria. Patients were monitored for adverse events. Study drugs were stopped if a drug-related SAE or AE of grade 4 or higher occurred, in case of AIDS-related illnesses CDC C, or if the blood CD4+ T cell count dropped below 200.

This was an open label study using approved drugs with a known safety profile for a shorter duration (pyrimethamine and valproic acid) than in usual care. The lab results (blood CD4+T-cells, plasma HIV RNA) and clinical condition that define the safety of this trial were readily available of all patients for the investigators. To protect the safety of the patients, the following study stopping rules were used:

1. A pre-specified interim analysis will be done after 14 patients (50%) are randomized. This will focus on the number of patients that had to discontinue treatment on investigators discretion due to blood CD4+T-cell count <200 during the IMP use, CDC-C events, possibly drug related SAE/AE ≥grade4, or AIDS related illness CDC C. If this exceeds 2 (10% of 21 patients that undergo an intervention) patients, the study will be stopped.
2. If ≥2 patients (of the 21 patients that undergo an intervention) have discontinued treatment due to possibly drug related AE before the planned interim analysis is done, the study will be stopped.
3. If at any time in the study the number of discontinuations due to blood CD4+T-cell count <200 during IMP use, or CDC-C events or possibly drug related SAE/AE ≥grade4, AIDS related illness CDC C exceeds 25% of 21 patients undergoing an intervention (>5 patients), the study will be stopped.
4. If ≥2 patients (10%) experience HIV treatment failure with acquisition of resistance associated mutations in HIV, the study will be stopped.
